# Supplementary material for: Assessing the impact of aggregating disease stage data in model predictions of human African trypanosomiasis transmission and control activities in Bandundu province (DRC)
Source: PLoS Negl Trop Dis. 2020 Jan 21;14(1):e0007976. doi: 10.1371/journal.pntd.0007976 (PMC6994134; doi:10.1371/journal.pntd.0007976)
Supplement: S1 Text — (PDF) [file pntd.0007976.s001.pdf]

S1 Text. Remarks on former Bandundu province case report data

Publicly available HAT case report data was used in this study from [1] and is shown below. Annual active screening covered approximately 7-13% of the estimated population between 2000 and 2012. The annual ratio of cases detected by active and passive varied between 0.8 and 1.9, and was greater than one in all years except 2000.

During the data analysis an error was reported in the published number of screened people in year 2008. After request to the corresponding author, the corrected values (875,469 for active screening and 69,945 for passive detection) were used, replacing the original values (702,190 and 243,179 respectively).

A small percentage (0.4-2.8%) of the data were unstaged. In the present study these unstaged cases were proportionally distributed among both stages for fits to staged and subset staged data.

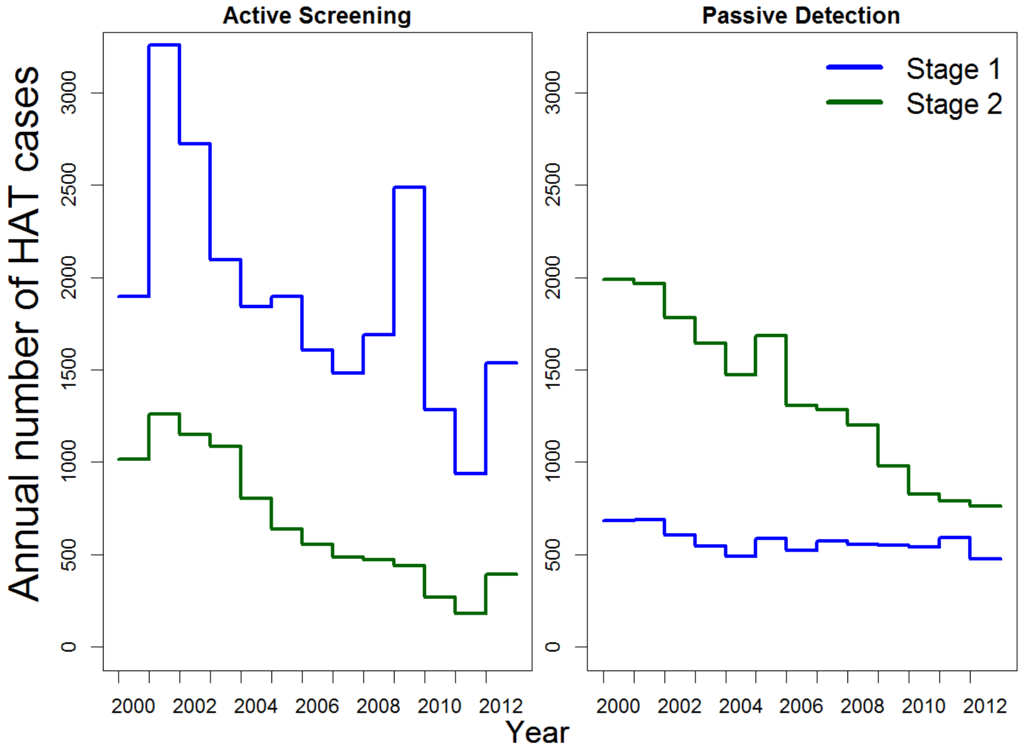

Reported cases for Bandundu province from active screening and passive detection, period 2000-2012.

References

1. Lumbala C, Simarro PP, Cecchi G, Paone M, Franco JR, Mesu VK, Makabuza J, Diarra A, Chansy S, Priotto G, Mattioli RC. Human African trypanosomiasis in the Democratic Republic of the Congo: disease distribution and risk. International journal of health geographics. 2015 Dec;14(1):20.
