# Supplementary material for: Assessing the impact of aggregating disease stage data in model predictions of human African trypanosomiasis transmission and control activities in Bandundu province (DRC)
Source: PLoS Negl Trop Dis. 2020 Jan 21;14(1):e0007976. doi: 10.1371/journal.pntd.0007976 (PMC6994134; doi:10.1371/journal.pntd.0007976)
Supplement: S2 Text — (PDF) [file pntd.0007976.s002.pdf]

S2 Text. Model descriptions

Model description for the four models used in the article *Assessing the impact of aggregating disease stage data in model predictions of human African trypanosomiasis transmission and control activities in Bandundu province (DRC)*. Code for each model is attached in a separate Supplementary Information file.

Model I

Transmission model

The Institute for Disease Modeling (IDM) model (Model I) is a deterministic compartmental model that captures the most prominent features of human African trypanosomiasis (HAT). A diagram of the model is given in Figure 1. It is a variant of the model used in a previous paper ([1]). This simple model structure allows for most of the parameters to be calibrated given the quantity of data available.

The key difference with the previous model is the inclusion of a refractory population in the tsetse to capture the teneral phenomenon of trypanosome infection of the vector. The two host stages are allowed to be dynamically different, where we allow the biting rate of tsetse flies on stage 1 and stage 2 humans to vary.

This captures potential differences in population level behaviour between the two stages. Hence, the division need not directly coincide with the transit of the blood brain barrier in the host, but rather with the point where the behavior of the host becomes sufficiently different to alter the interactions with tsetse.

With this in mind, we included the stage transition rate among the parameters that we allowed to vary for calibration, although the prior for this parameter was tightly controlled.

The other parameters which we allowed to vary for the calibration were stage dependent disease induced mortality, transmission parameters with two stage dependent host to vector transmission rates, and one vector to host transmission rate, stage dependent active and passive screening rates, rate of return to the susceptible class after treatment, stage dependent initial prevalence, initial recovered proportion, initial vector prevalence, and initial proportion of vectors that were refractory for a total of 16 calibrated parameters. The choice to fit the initial conditions was based on the lack of data for previous interventions.

The remaining values needed for the model were birth and death rates of non-infected individuals, death rate of vectors, and the refractory rate for the vectors. These values, with the exception of the birth rate, were chosen from literature. The birth rate was set so as to produce a 3% annual growth of the population.

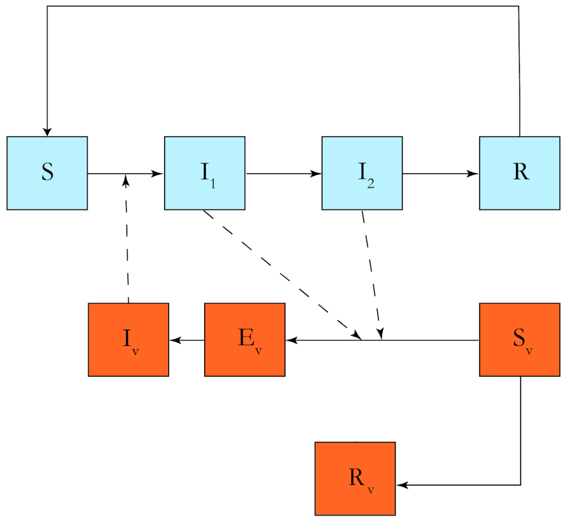

Figure 1: Schematic for Model I. The blue boxes represent the host and the orange boxes represent the vectors. The vectors have a refractory compartment, R_v, to account for the teneral phenomenon.

Fitting methodology

A Bayesian approach was used to calibrate the model to each subset of data. The choice was made to use a Bayesian approach since it was capable of simply accounting for the uncertainty in the observation process (active and passive screening). Hence, we need only to reconstruct the observation process in the model and compare it against the distribution associated with the data, rather than directly measure the effect of the model dynamics on parameter uncertainty (which would require an approximate Bayesian computation (ABC) approach, since an analytical approach would require closed form solutions of the system of differential equations). For this model, this method represented the most direct approach to the problem.

We now give a description of how we modelled the observation process and derived the associated likelihoods for the Bayesian computations. Active screening was considered on a daily basis, where the number of people screened each day was the daily average of people screened for that year (that is, the total number of people screened, divided by 365). The number of cases found per day was considered to be a realization of a binomial random variable with probability determined by the prevalence in the model dynamics for that given day. The daily cases were summed to produce the yearly totals. However since, the sum of binomial random variables

has an intractable probability mass function (pmf), we approximated the daily number of cases with a Poisson random variable whose parameter is the product of the number of people screened daily and the daily prevalence (this is justified since the prevalence is small); and since the sum of Poisson random variables is Poisson, we used a Poisson pmf for the likelihood for the yearly cases. For simplicity, the number of cases each year were considered to be independent, so that the total likelihood for the Bayesian approach was the product of the various Poisson pmfs for the years.

Naive Markov Chain Monte Carlo (MCMC) sampling of the prior parameter space had prohibitively large rejection rates, and so a modified sampling scheme was used that approximates the distributions of the likely solutions. Several local maxima of the likelihood were found through traditional optimisation, begun at randomly selected points within the prior space. We then sample around these local maximum likelihood estimators (MLEs) with multivariate normal distributions. The new samples were accepted with probability equal to the ratio of the likelihood value with the MLE. The variance of these normal distributions were chosen so that the acceptance rate was approximately 20%, so that the new samples were sufficiently different for the local MLE we were sampling around. This targeted sampling method improved our efficiency in sampling from feasible regions of the parameter space.

Due to the first optimisation step in our sampling approach, we were able to begin with relatively large prior distributions for the calibrated parameters. These, and the values for the fixed parameters, are listed in Table 1 (note that since we are calibrating parameters that are coefficients of dimensionless variables in the ordinary differential equations, they all have units $1/\text{days}$).

**Parameter**	**Units**	**Prior**
Stage Transition Rate	1/days	Unif$(.0015, .005)$
Stage 1 Death Rate	1/days	Unif$(.000054795, .0001)$
Stage 2 Death Rate	1/days	Unif$(.0015, .005)$
Vector to Host Transmission	1/days	Unif$(0, 10)$
Stage 1 Passive Screening Rate	1/days	Unif$(.5, 5)$
Stage 1 Active Screening Rate	1/days	Unif$(1, 5)$
Stage 2 Passive Screening Rate	1/days	Unif$(0, 10)$
Stage 1 Host to Vector Transmission	1/days	Unif$(0, 1)$
Stage 2 Host to Vector Transmission	1/days	Unif$(0, 1)$
Stage 2 Active Screening Rate	1/days	Unif$(0, 10)$
Return to Susceptible Rate	1/days	Unif$(0, .03)$
Initial Stage 1 Prevalence	-	Unif$(0, .01)$
Initial Stage 2 Prevalence	-	Unif$(0, .01)$
Initial Recovered Proportion	-	Unif$(0, .1)$
Initial Infected Vector Proportion	-	Unif$(0, .1)$
Initial Refractory Vector Proportion	-	Unif$(.5, .9)$
Susceptible Birthrate	1/days	$\ln(1.03/365)$
Vector Death Rate	1/days	.03 [2]
Vector Refractory Rate	1/days	.33 [2]

Table 1: Parameterisation for Model I.

Priors come either directly from the previous study, or are up to 50% expansions (within reasonable values). Given the targeted sampling scheme implemented, and the very narrow peaks in the likelihood, posteriors were not particularly sensitive to the choice of bounds on these values. Posteriors are plotted as histograms in Figures 2–4.

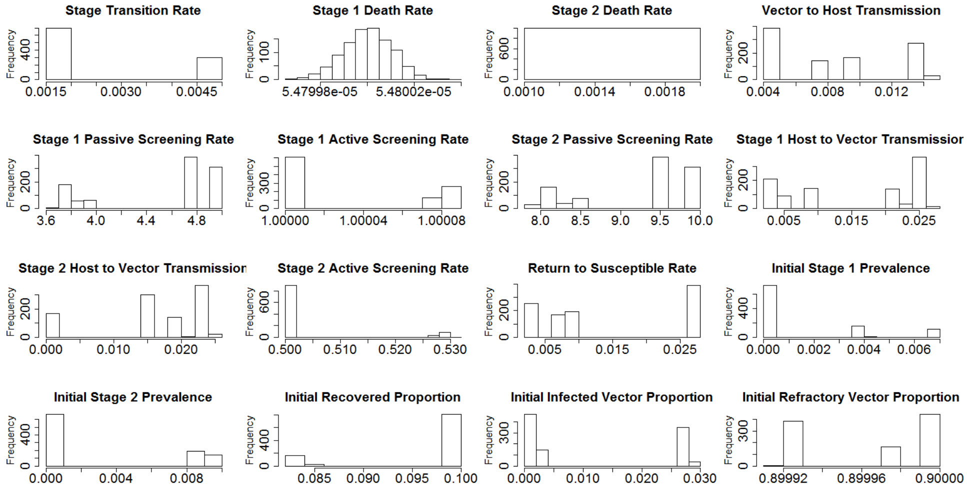

Figure 2: Posteriors for Fit A for Model I.

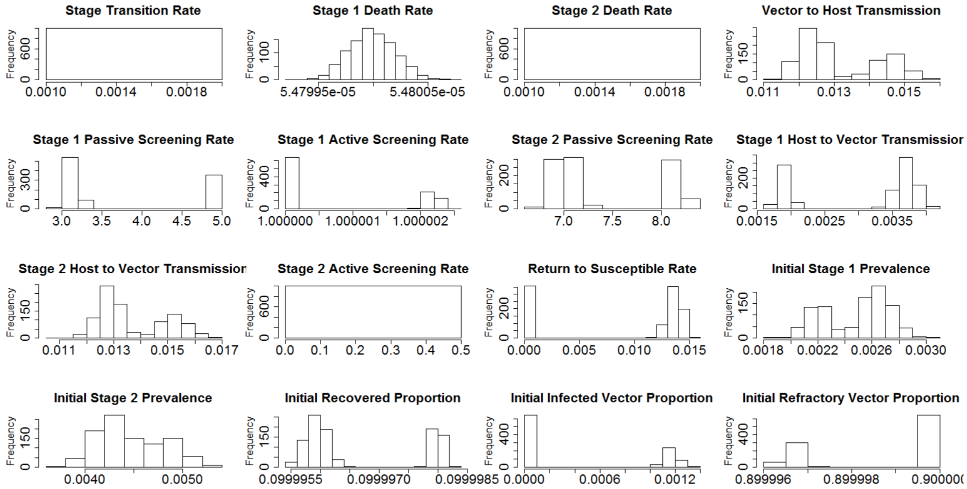

Figure 3: Posteriors for Fit B for Model I.

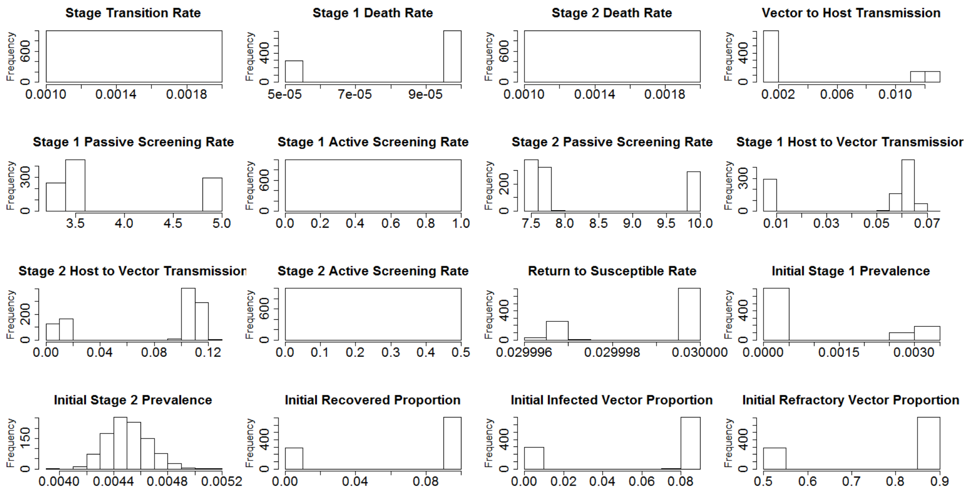

Figure 4: Posteriors for Fit C for Model I.

Model S

Transmission model

The Swiss TPH model (Model S) is a variant of the HAT transmission model published in [3], consisting of a system of coupled ordinary differential equations. The deterministic model consists of compartments for tsetse, animal and human populations. These three different host types are modelled for two different settings corresponding to a low transmission area (e.g. the village, L) and a high transmission area (such as river banks or plantations, H) that enable accounting for heterogeneity in exposure to tsetse bites. As previously, the population size for tsetse, animal or humans in each setting i ($i = \{L, H\}$) is assumed to be stable by allowing the associated birth terms to compensate deaths in all the compartments. Tsetse and animal populations always stay within their setting (for example, tsetse in low transmission settings always remain in the low transmission setting and animals in high transmission settings always remain in the high transmission setting). Similarly, humans in low transmission settings always remain in low transmission setting. However, humans in the high transmission setting move back and forth between the high and low transmission settings spending a fixed amount of time in each one (to model, for example, the movement of high risk individuals between villages and plantations) — as shown in Figure 5.

Five compartments describe humans in any of the two settings: susceptible (S_{hi}); exposed or incubating (E_{hi}); infected with the first stage of the disease (I_{h1i});

infected with the second stage of the disease, where trypanosomes have reached the cerebro-spinal fluid (I_{h2i}); and treated (T_{hi}). The total human population in setting i is $N_{hi} = S_{hi} + E_{hi} + I_{h1i} + I_{h2i} + T_{hi}$.

Tsetse populations are divided into susceptible (S_{vi}); teneral (U_{vi}); exposed (E_{vi}); and infected (I_{vi}), so that the vector population is $N_{vi} = S_{vi}+U_{vi}+E_{vi}+I_{vi}$.

In this version of the HAT model, we assume animals do not contribute to transmission, thus animal populations are modelled as constant parameters, N_{ai}, and only form a sink for tsetse bite.

Assumptions for HAT transmission that are new to this variant of the model are the following: (a) both stages of the disease are exposed to tsetse fly bites (in the previous version, only stage 1 was exposed to tsetse flies); (b) a compartment indicated with U_i was added to the vector dynamics to account for the teneral effect — a reduction of infectivity with time — such that on average tsetse are only infectious for the first five days after emergence. These changes were made to provide a more realistic representation of the transmission dynamics.

Test and treat interventions encompass both active screening and passive surveillance. Passive detection is represented by a continuous stage-specific detection rate and removes infected people from both low- and high-risk settings whilst active screening only recruits people in the low risk setting.

Active screening was modelled as as a pulsed activity taking place in the first month of each year as this is a more realistic representation of actual campaigns than the continuous rate of active screening used previously in this model [3]. We followed [4] to relate a proportion, d, of humans effectively screened in a given a year and the daily removal rate $r_{as}^{\text{continuous}}$ as $d = 1 - \mathrm{e}^{-365 r_{as}^{\text{continous}}}$. Thus, for the pulsed active screening, we get: $r_{as}^{\text{pulsed}} = 12 r_{as}^{\text{continous}} = -(12/365)\ln(1-d)$. Screening levels were informed from data; estimates for the population of Bandundu were taken from [5] for the period corresponding to each calibration, and a 3% annual growth was assumed for projections.

We included a new parameter, ϵ, to represent the unknown proportion of the population at risk of infection in Bandundu province, such that $d(t) = \frac{X_s(t)}{\epsilon N_B(t}$, where $X_s(t)$ indicates number of people screened in year t, and $N_B(t)$ indicates Bandundu province population in year t. With no additional data enabling estimating ϵ, this parameter was set as a constant value in all of the three different fits performed.

A schematic of the model is shown in Figure 5, while model parameters are described in Table 2 . Fixed parameters values were taken from Model S posteriors (median) in [1] unless indicated otherwise.

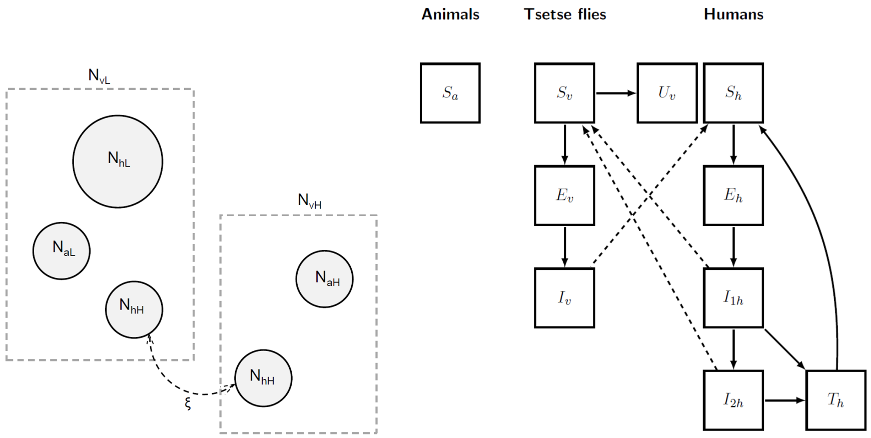

Figure 5: Schematic of Model S. Left: model population structure. Human populations are composed by a stationary population (N_{hL}) that remains in low exposure habitats (e.g., a village), and a smaller population (N_{hH}) which commute and spend a proportion ξ of their time in a potentially high exposure setting (e.g., a plantation). Each habitat also contains tsetse (N_{vL} and N_{vH}) and non-human vertebrate animal populations (N_{aL} and N_{aH}). Right: schematic of infection dynamics, subscripts $i = \{L, H\}$ were removed for easy reading. Compartmental diagram highlights the transmissions between states of infection of the tsetse and human populations, with solid lines indicating transition between compartments, and dashed lines representing transmission rates. Animals cannot transmit infection thus acting as a sink for tsetse bite. Note that in the low-risk transmission setting, both human populations are exposed to tsetse bites. Figure adapted from [3].

Notation	Description	Unit	Value
α	Rate at which tsetse become non-teneral (i.e. cannot get infectious)	Year^{-1}	73
A/H_1	Density of animals relative to humans in area L	-	0.7
A/H_2	Density of animals relative to humans in area H	-	0.9
b	Proportion of infective bites leading to infection in humans and animals	-	0.8
c_h	Proportion of bites on an infective human that lead to a mature infection in flies	-	fitted
c_{ai}	Proportion of bites on an infective animal of type i that lead to a mature infection in flies	-	0
δ	Rate at which treated humans return to the susceptible class	Year^{-1}	2.19
δ_a	Rate of loss of immunity in animal hosts	Year^{-1}	1.095
ϵ	Proportion of population at risk	-	0.7
η	Rate at which hosts move from the incubating stage	Year^{-1}	31.025
f	Inverse of duration of feeding cycle; or biting rate	Year^{-1}	121.545
γ	Rate of progression to stage 2 in humans	Year^{-1}	0.365
γ_{aL}	Rate of progression to the immune class in animal hosts of type L	Year^{-1}	0.73
γ_{aH}	Rate of progression to the immune class in animal hosts of type H	Year^{-1}	0.6935
μ	Death rate of humans due to natural causes	Year^{-1}	0.02
μ_{ai}	Death rate of animal host of type i	Year^{-1}	0.511
μ_γ	Disease-induced death rate or rate of leaving the recovered state for humans	Year^{-1}	fitted
μ_t	Death rate of humans due to treatment	Year^{-1}	0
μ_v	Death rate of tsetse	Year^{-1}	10.95
ν	Inverse of the extrinsic incubation period	Year^{-1}	13.505
m_{HL}	Ratio of humans in the high exposure to low exposure environment	-	fitted
r_1	Removal rate of infected humans in stage 1 due to treatment (passive detection)	Year^{-1}	4.6144
r_2	Removal rate of infected humans in stage 2 due to treatment (passive detection)	Year^{-1}	fitted
σ	Biting preference for humans	-	0.4
σ_{ai}	Biting preference for animal in the setting i	-	0.3
vh_i	Number of vectors per human in area i	-	fitted
ξ	Proportion of time spent i the high risk region by commuters	-	0.62

Table 2: Parameterisation for Model S. Here $i = \{L, H\}$. The human population sizes, N_{hi} are calculated from m_{HL}. The animal populations, N_{ai}, are products of the animal densities relative to humans and the human populations; and the tsetse populations, N_{vi}, are products of the tsetse densities relative to humans and the human populations.

Fitting procedure

The model was run to reach equilibrium prevalence of infection prior to fitting assuming on-going passive screening only. The model was calibrated to the three different data configurations described in the main text via approximate Bayesian computation (ABC) fitting six parameters (human infectivity, c_h; passive detection rate of stage 2, r_2 (through a constant of proportionality, c_2); stage 2 human mortality rate, μ_γ; proportion of humans in high- to low-risk setting, m_{HL}; and proportion of vector to human in both settings, vh_L and vh_H). In all cases uniform prior distributions were used (Table 3).

For the fits to staged data, an improvement in passive detection was included in the detection rate of stage 2, r_2, by multiplying the fitted constant of proportionality, c_2, by the proportion of people screened through passive surveillance as informed by data which showed an increasing trend.

Annual reported cases distinguished by detection strategy (active or passive) was used as summary statistics in the ABC procedure. Reported cases were additionally divided by stage in the two fits using staged data. Parameter sets were rejected if simulations for those sets did not satisfy the error tolerance,

$$\max_{d,s,y} \left| \frac{D_y^{d,s} - R_y^{d,s}}{D_y^{d,s}} \right| < 0.4,$$

where $D_y^{d,s}$ denotes the number of reported cases from the data and $R_y^{d,s}$ denotes the number of reported cases from the model separated by detection method, d (active or passive) for all three data sets; disease stage, s (1 or 2) for the staged and subset staged data sets; and year of detection, y (2000–2012 for the staged and unstaged data sets and 2000–2006 for the subset staged data set). The trend in the number of stage 1 reported cases by passive detection for Bandundu province (see "S1 Text. Remarks") is decreasing. Using error tolerances $> 40\%$ systematically led to accepting a high proportion of simulations with an increasing trend for the number of stage 1 cases, which is the opposite of the observed trend in this data set. Therefore, the choice of an error tolerance of 40% enabled an optimised search in the parameter space by rejecting most of these cases.

Summary statistics for the posterior distribution of parameters estimated for the three different fits is shown in Table 4 and the posterior distributions are shown in Figures 6–8.

Parameter	Description	Unit	Prior
c_h	Proportion of bites on an infective human that lead to a mature infection in flies	-	$X \sim$ Unif$[.001, .004]$
c_2	Constant of proportionality relating proportion of population screened to detection rate	Year^{-1}	$X \sim$ Unif$[0, 80]$
μ_γ	Disease-induced death rate or rate of leaving the recovered state for humans	Year^{-1}	$X \sim$ Unif$[0.42, 0.73]$
m_{HL}	Ratio of humans in the high exposure to low exposure environment	-	$X \sim$ Unif$[0.001, 0.3]$
vh_L	Number of vectors per human in area L	-	$X \sim$ Unif$[2, 4]$
vh_H	Number of vectors per human in area H	-	$X \sim$ Unif$[2, 4.5]$

Table 3: Priors for fitted parameters for Model S.

Parameter	Fit A		Fit B		Fit C	
	Median	95% CI	Median	95% CI	Median	95% CI

Table 4: Summary statistics of the posterior distributions for parameters for Model S.

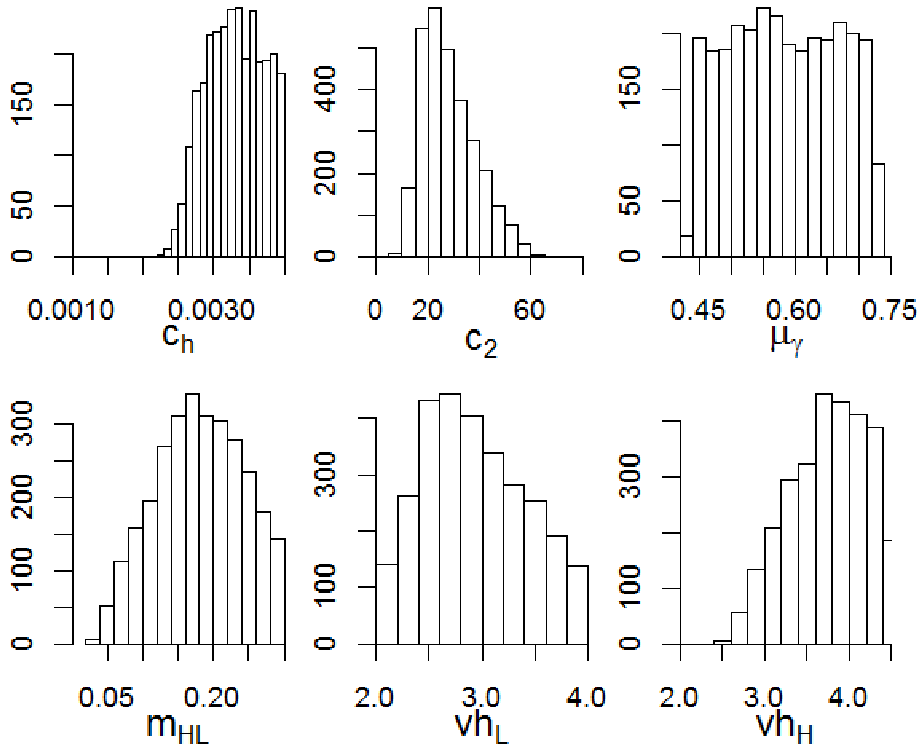

Figure 6: Posterior parameter distributions for Fit A (unstaged data) for Model S.

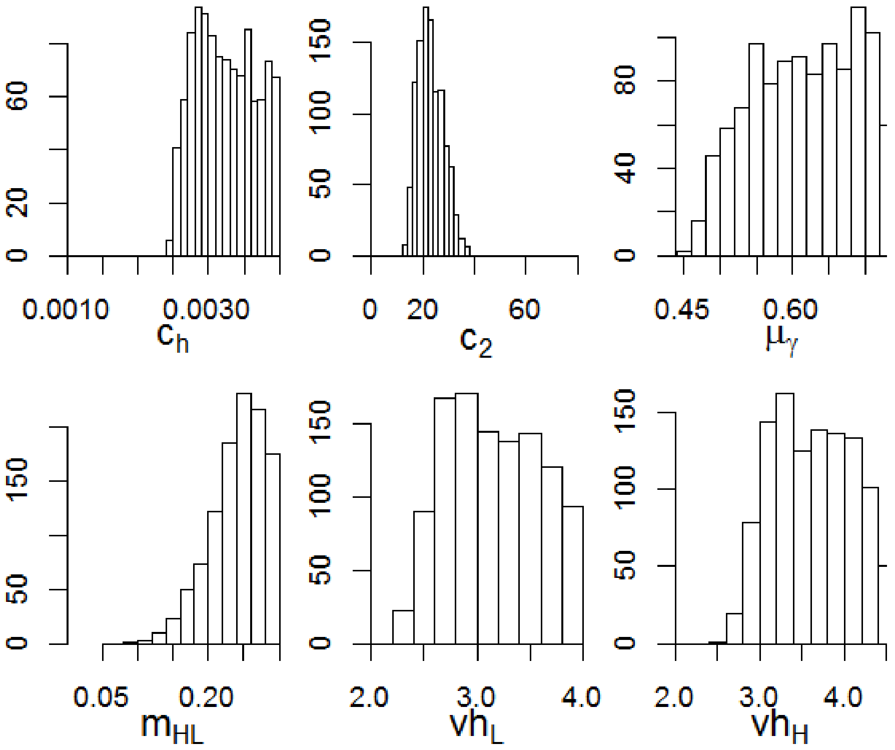

Figure 7: Posterior parameter distributions for Fit B (staged data) for Model S.

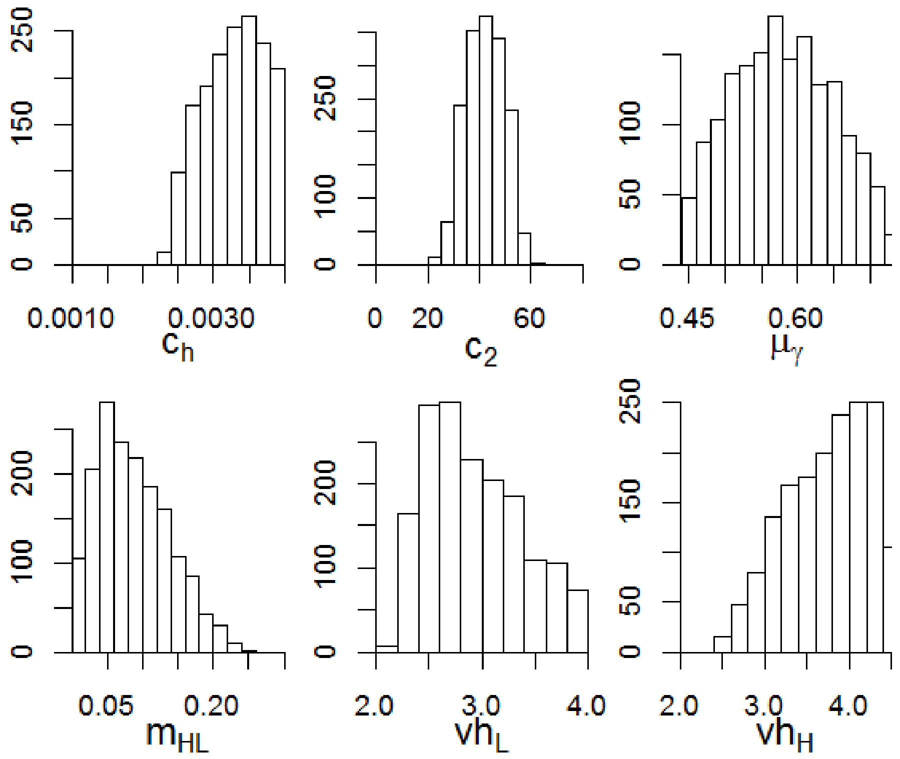

Figure 8: Posterior parameter distributions for Fit C (7 years staged data) for Model S.

Model W

Transmission model

The Warwick HAT model (Model W) and its variations have been described in previous publications [1, 6–9]. The version used in the present study is a compartmental ODE model which describes the disease dynamics of human and tsetse populations (Figure 9). Following previous model selection studies [6, 9], humans are considered to either be at low- or high-risk of exposure to tsetse, with a higher biting pressure on high-risk humans. Tsetse are also assumed to feed on other non-reservoir animals, which cannot transmit to tsetse. In the present study, the role of animal reservoirs is not considered.

Upon infection from tsetse, humans pass through a brief incubation period (E_H), and then enter stage 1 disease (I_{1H}). During this stage, there is only a low passive detection rate, d_1, as individuals with non-specific symptoms may not be picked up through standard passive detection. After a period of $1/\varphi_H$ days on average, undetected people develop stage 2 disease (I_{2H}). The passive detection rate is given by $u\gamma_H$ for stage 2 infection, which has more specific symptoms; this rate combines the exit rate for stage 2 (treatment or death) and the reporting probability (u). Stage 1 and 2 infected people are assumed to be equality infectious to tsetse. Typical active screening (denoted by ν_H in Figure 9) is assumed to only recruit people from the low-risk group and has a full diagnostic algorithm sensitivity of 91%. Screening is assumed to occur annually. After active or passive diagnosis, patients are treated in hospital (R_H) and recover at home become returning to a susceptible status after a period $1/\omega_H$.

Tsetse population dynamics are modelled explicitly including a pupal stage (P_V), teneral/unfed adults which are susceptible to infection (S_V), non-teneral and uninfected adults which have significantly reduced (95%) susceptibility to infection (G_V), tsetse incubating infection (E_V) and tsetse with mature salivary gland infections (I_V).

To simulate the impact of intensified interventions, some additional modifications were made:

- To model the impact of tsetse control it was assumed that tiny targets were deployed at six-monthly intervals. The probability of a tsetse hitting a target and dying during the blood-feeding phase of the cycle is given by:

$$f_T(t) = f_{\text{max}} \left(1 - \frac{1}{1 + \exp(-0.068(\text{mod}(t, 182.5) - 127.75))}\right) \quad (1)$$

  with f_{max} representing the maximum target efficacy just after initial deployment following Rock *et al* [8].

- To simulate the impact of enhanced passive detection the reporting probability, u, may be scaled to so that $u \mapsto (1+u)/2$ (i.e., the probability of underreporting is halved). For the same year, the two rate parameters corresponding to passive detection from stage 1 and exiting stage 2, d_1 and γ_H respectively, were doubled. This strategy is the same used previously [1], and it is noted (once again) that the most appropriate parameter increase in the model to capture the real-world impact enhanced passive detection remains unclear.

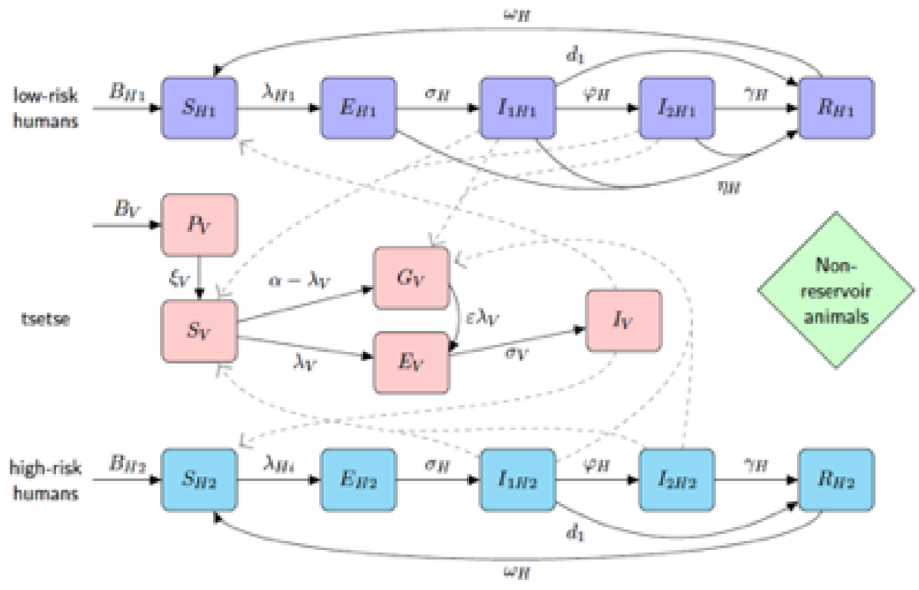

Figure 9: Schematic of Model W. Boxes show compartments representing different types of hosts (blue and purple) and vectors (pink), and disease status and progression for each host/vector type (solid lines). Gray lines show possible routes of transmission between tsetse and humans. This figure is reproduced from [1].

Fitting procedure

The model was fitted three times, to the unstaged (Fit A), staged (Fit B) and subset staged (Fit C) data described in the main text. Eight parameters, R_0, r, k_1, d_1, u, γ_H, disp$_{act}$, and disp$_{pass}$, were fitted for all three data sets. Descriptions and values of all fixed parameters are given in Table 5 and descriptions and prior distributions

for all fitted parameters are given in Table 6. In the fit to 13 years staged data (Fit B) an additional parameter, d_{amp} was fitted. This additional parameter, which did not feature in previously published iterations of Model W, was used to fit to the increase in the percentage of stage 1 reporting in passive detection by increasing the stage 1 passive detection rate, d, such that passive stage 1 reporting is described by:

$$d(Y) = d_1 \left[1 + \frac{d_{\text{amp}}}{1 + \exp\left(-d_{\text{steep}}(Y - d_{\text{change}})\right)} \right]. \tag{2}$$

This was not used for the other fits as there was no observable change in the data — either there was no staging information (Fit A), or there were too few years (Fit C) to detect an increase in the stage 1 to 2 passive case ratio (the change occurs post 2006).

Table 5: Parameterisation (fixed parameters) for Model W

Notation	Description	Value	
N_H	Total human population size	9.0744×10^6	
B_H	Total human birth rate	$= \mu_H N_H$	
μ_H	Natural human mortality rate	5.4795×10^{-5} days^{-1}	[10]
σ_H	Human incubation rate	0.0833 days^{-1}	[11]
φ_H	Stage 1 to 2 progression rate	0.0019 days^{-1}	[12, 13]
ω_H	Recovery rate/waning-immunity rate	0.006 days^{-1}	[14]
Sens	Active screening diagnostic sensitivity	0.91	[15]
Spec	Active screening diagnostic specificity	0.999	[15]
B_V	Tsetse birth rate	0.0505*	[8]
ξ_V	Pupal death rate	0.037 days $^{-1}$	
K	Pupal carrying capacity	$= 111.09 N_H^{\dagger}$	[8]
$\mathbb{P}$(pupating)	Probability of pupating	0.75	
μ_V	Tsetse mortality rate	0.03 days^{-1}	[11]
σ_V	Tsetse incubation rate	0.034 days^{-1}	[16, 17]
α	Tsetse bite rate	0.333 days^{-1}	[18]
p_V	Probability of tsetse infection per single infective bite	0.065	[11]
ε	Reduced non-teneral susceptibility factor	0.05	[6]
f_H	Proportion of blood-meals on humans	0.09	[19]
f_{max}	Probability of both hitting a tiny target and subsequently dying at time t (60% reduction after 1 year)	0.0302	
d_{steep}	Steepness of change in S1 passive detection function	1	Assumed
d_{change}	Switching point in S1 passive detection function	2008	Assumed

* Value is chosen to maintain constant population size without interventions (see [8]).

† Value is chosen to reflect the observed bounce back rate (see [8]).

Table 6: Parameterisation (fitted parameters) for Model W.

Notation	Description	Unit	Prior
R_0	Basic reproduction number (Next Generation Matrix approach)	-	$X \sim$ Unif$[0, \infty)$
d_1	Treatment rate from stage 1	Days^{-1}	$X \sim$ Unif$[0, \text{inf})$
γ_H	Treatment rate from stage 2	Days^{-1}	$X \sim \mathcal{N}(0.0045, 6.4 \times 10^{-7})$
u	Proportion of passive cases reported	-	$X \sim B(5, 5)$
k_1	Proportion of low-risk people	-	$X \sim$ Unif$[0, 1]$
k_4	Proportion of high-risk people	-	$k_4 = 1 - k_1$
m_{eff}	Effective tsetse density	-	Fitted as part of R_0 as $R_0^2 \propto m_{eff}$
p_H	Probability of human infection per single infective bite	-	Fitted as part of R_0, (N.B. $m_{eff} = N_V p_H / N_H$)
N_V	Tsetse population size	-	Fitted as part of R_0, (N.B. $m_{eff} = N_V p_H / N_H$)
r	Relative bites taken on high-risk humans	-	$X \sim$ Unif$[1, 100]$
disp_{act}	Overdispersion for active screening	-	$X \sim$ Unif$[1, \infty)$
$\text{disp}_{\text{pass}}$	Overdispersion for passive screening	-	$X \sim$ Unif$[1, \infty)$
$d$amp	Amplitude of change in S1 passive detection function	-	$X \sim$ Unif$[0, 3]$

The adaptive Metropolis-Hastings MCMC used in this study took the log-likelihood function to be:

$$\begin{aligned} LL(\theta|x) = & \log(P(x|\theta)) \\ \propto & \sum_{i=2000}^{2007 \text{ or } 2012} \Bigg(\log \left[\text{BetaBin} \left(A_{D1}(i) + A_{D2}(i); z(i), \frac{A_{M1}(i) + A_{M2}(i)}{N_H}, \text{disp}_{\text{act}} \right) \right] \\ & + \log \left[\text{Bin} \left(A_{D1}(i); A_{D1}(i) + A_{D2}(i), \frac{A_{M1}(i)}{A_{M1}(i) + A_{M2}(i)} \right) \right] \\ & + \log \left[\text{BetaBin} \left(P_{D1}(i) + P_{D2}(i); N_H, \frac{P_{M1}(i) + P_{M2}(i)}{N_H}, \text{disp}_{\text{pass}} \right) \right] \\ & + \log \left[\text{Bin} \left(P_{D1}(i); P_{D1}(i) + P_{D2}(i), \frac{P_{M1}(i)}{P_{M1}(i) + P_{M2}(i)} \right) \right] \Bigg) \end{aligned}$$

In the "unstaged" case, j denotes all reported cases of that type, whereas in the two "staged" fits, the $j \in \{1, 2\}$ denotes for stage 1 and stage 2 cases respectively. The model takes parameterisation θ, x is the data, $P_{Dj}(i)$ and $A_{Dj}(i)$ are the number of passive/active cases (of stage j) in year i of the data, $P_{Mj}(i)$ and $A_{Mj}(i)$ are the number of passive/active cases (of stage j) in year i of the model, and $z(i)$ is the number of people screened in year i. Beta-Bin$(m; n, p, a)$ gives the probability of obtaining m successes out of n trials with probability p and overdispersion parameter a. The overdispersion accounts for larger variance than under the binomial; this feature of the model fitting was new compared to previous fits for health zones which were performed using binomial pdfs. The tight credible intervals of the binomial became particularly pronounced at the provincial scale and so the Beta-Binomial was deemed more appropriate for capturing sampling uncertainty. N.B. The pdf of this Beta-Binomial distribution is given by:

$\text{BetaBin}(m; n, p, \rho)$ gives the probability of obtaining m successes out of n trials with probability p and overdispersion parameter ρ (which is given by the value disp_{act} or $\text{disp}_{\text{pass}}$ for active and passive screening respectively). The overdispersion accounts for larger variance than under the binomial. The pdf of this distribution is given by:

$$\text{BetaBin}(m; n, p, \rho) = \frac{\Gamma(n+1)\Gamma(m+a)\Gamma(n-m+b)\Gamma(a+b)}{\Gamma(n-m+1)\Gamma(n+a+b)\Gamma(a)\Gamma(b)}$$

where $a = p(1/\rho - 1)$ and $b = a(1-p)/p$.

Results

For each of the three fits, a burn-in of 10,000 steps was used, followed by 100,000 MCMC steps to generate posterior distributions. 1000 samples were taken from each, and 10 realisations were sampled from each parameter set to yield 10,000 samples to use for projections of different strategies.

Table 7 gives the summary information for fitted parameters, whilst Figures 10, 11 and 12 show the posterior parameter distributions for each of the three fits.

Summary

The key differences between the present study and previous versions of Model W and its fitting methodologies are:

- The use of an increasing stage 1 passive detection rate, $d(Y)$, in Fit B. Previously Model W had a fixed stage 1 detection rate over time [9] (and indeed it remained fixed here in Fits A and C, as the data were insufficiently informative to justify a varying parameter).
- Switching from a binomial log-likelihood function to beta-binomial to increase variation due to sampling bias for this provincial scale analysis.
- Slight change in the log-likelihood formulation to account for staged and unstaged data.

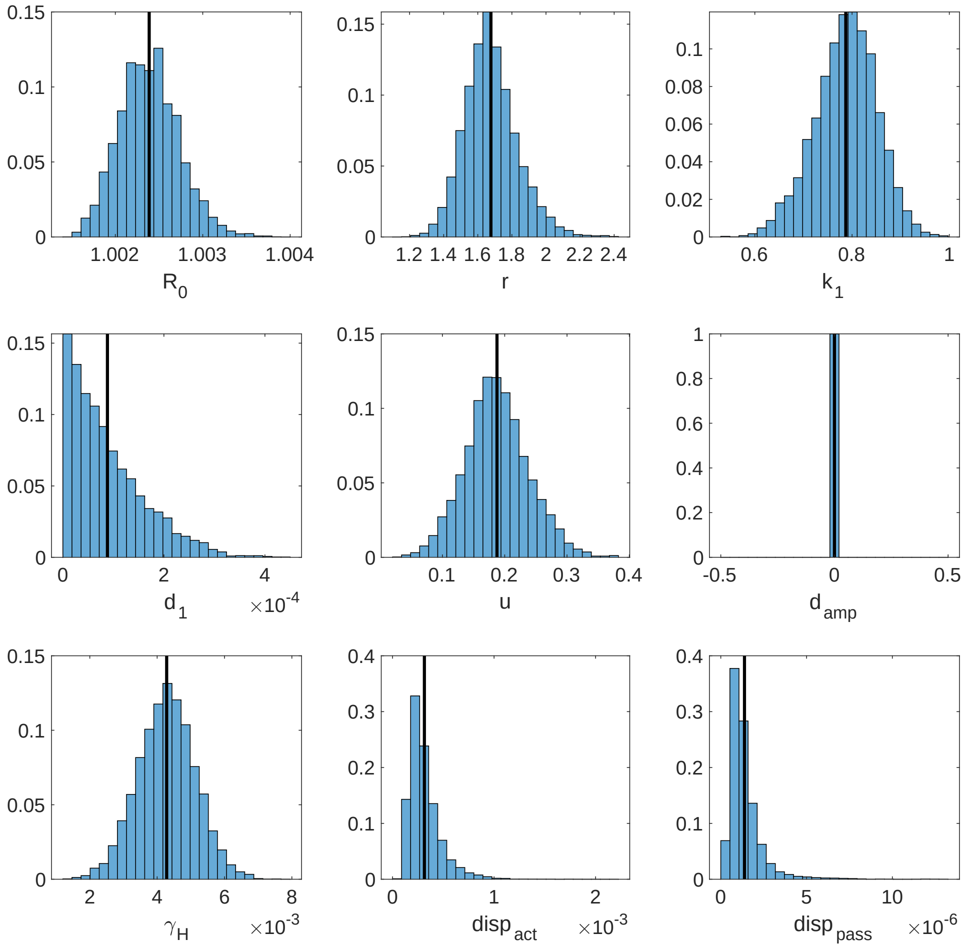

Figure 10: Posterior parameter distributions for Fit A (13 years unstaged data) for Model W.

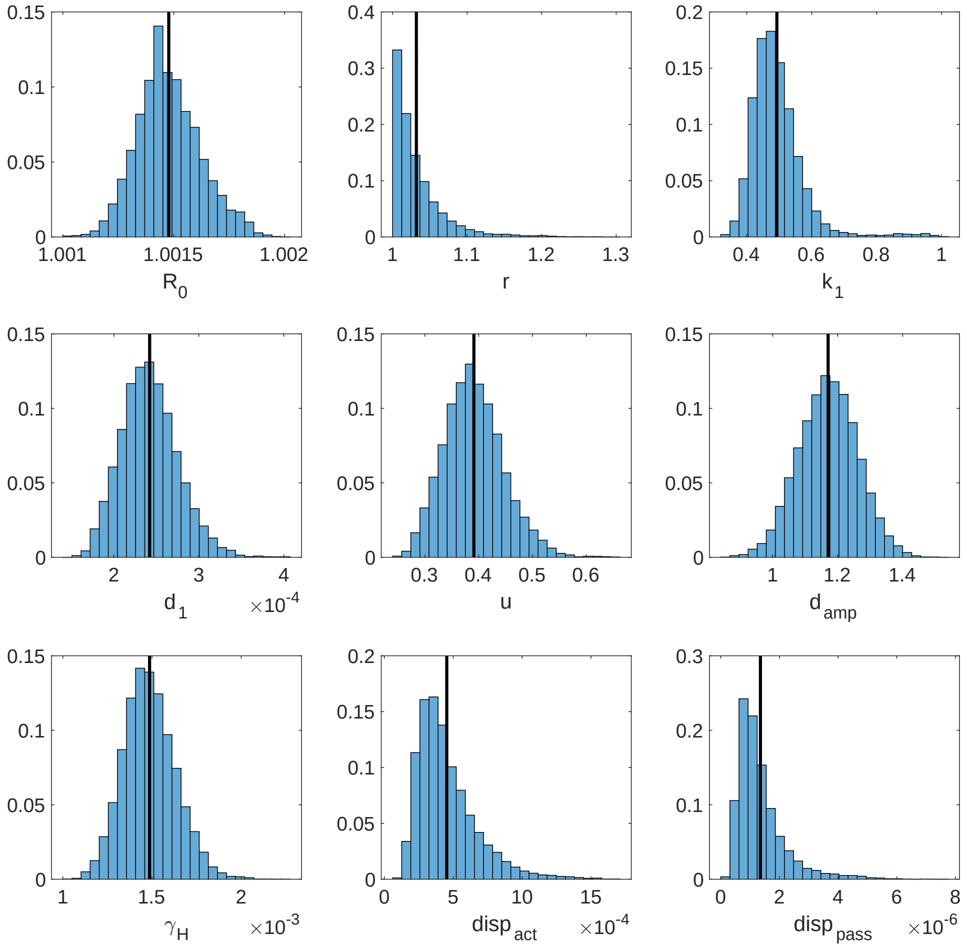

Figure 11: Posterior parameter distributions for Fit B (13 years staged data) for Model W.

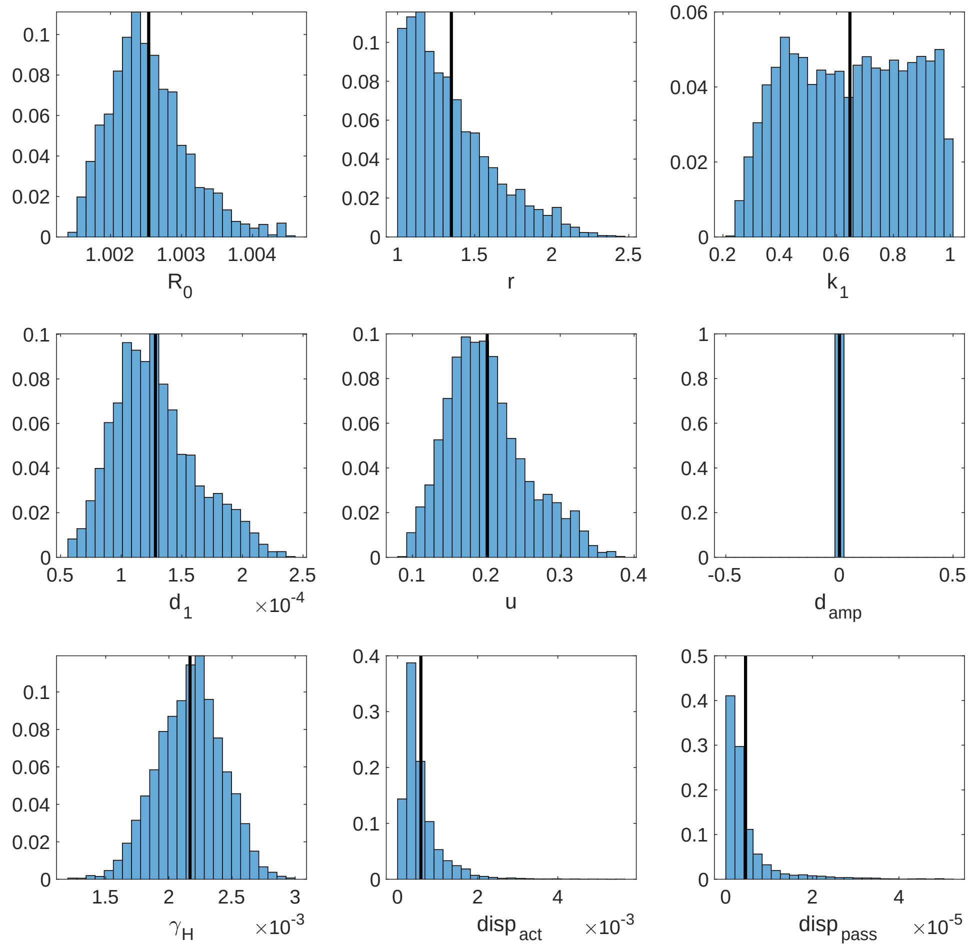

Figure 12: Posterior parameter distributions for Fit C (7 years staged data) for Model W.

	Fit A		Fit B		Fit C	
	Mean	95% CI	Mean	95% CI	Mean	95% CI
R_0	1.0024	[1.0018, 1.0031]	1.0015	[1.0012, 1.0018]	1.0025	[1.0017, 1.0038]
d_1 [Days^{-1}]	8.82×10^{-5}	[$2.65\times10^{-6}, 2.70 \times 10^{-4}$]	2.42×10^{-4}	[$1.83\times10^{-4}, 3.13 \times 10^{-4}$]	1.28×10^{-4}	[$7.22\times10^{-5}, 2.04 \times 10^{-4}$]
γ_H [Days^{-1}]	0.0043	[0.0026, 0.0060]	0.0015	[0.0012, 0.0018]	0.0022	[0.0017, 0.0027]
u	0.1876	[0.0912, 0.2917]	0.3911	[0.2934, 0.5097]	0.2012	[0.1130, 0.3241]
k_1	0.7871	[0.6522, 0.9042]	0.4927	[0.3826, 0.6877]	0.6469	[0.2994, 0.9788]
r	1.6770	[1.4001, 2.0166]	1.0319	[1.0008, 1.1305]	1.3488	[1.0147, 2.0401]
disp_{act}	0.0003	[0.0001, 0.0007]	0.0005	[0.0002, 0.0010]	0.0006	[0.0001, 0.0018]
$\text{disp}_{\text{pass}}$	1.38×10^{-6}	[$4.26\times10^{-7}, 3.99 \times 10^{-6}$]	1.35×10^{-6}	[$4.38\times10^{-7}, 3.79 \times 10^{-6}$]	4.56×10^{-6}	[$6.31\times10^{-7}, 2.38 \times 10^{-5}$]
d_{amp}	0.0000	[0.0000, 0.0000]	1.1705	[0.9926, 1.3463]	0.0000	[0.0000, 0.0000]

Table 7: Posterior parameter means and 95% credible intervals for Model W.

Model Y

Transmission model

The Yale model (Model Y) is a deterministic vector-host model for disease transmission between tsetse and human populations. The model implicitly accounts for non-reservoir animals through a biting preference parameter, which ensures that all tsetse bites are not directed to humans. This model was used in a previous publication ([8]).

We assumed that tsetse flies are susceptible to trypanosome infection only during their first blood meal and only within 24 hours after emergence from pupa to the adult stage. Susceptible adult tsetse flies become infected after feeding on an infectious human and enter the exposed state where the infection incubates. After incubation, tsetse flies become infectious for the rest of their life.

Humans may become exposed to infection after being bitten by an infectious tsetse. After the incubation period, a proportion of infected human hosts become symptomatic and enter stage 1 of the disease (Figure 13). Infected symptomatic human hosts would progress from stage 1 to stage 2 of infection, characterised by the severity of disease symptoms. The remaining proportion of infected human hosts do not become symptomatic and move to an asymptomatic compartment. We assume that these asymptomatic carriers never become symptomatic and recover after an average duration of infection (that was fitted to the data).

Contrary to the previous use of this model where we assumed differential infectivity between asymptomatic carriers and stages 1 and 2, here we assume that stage 1 and 2 and asymptomatic infections are equally infectious. Stage 1 patients either seek treatment and recover or progress to stage 2 due to being untreated or due to treatment failure. Stage 2 patients either seek treatment and recover or die due to being untreated or due to treatment failure. Successfully treated HAT patients are temporarily immune to reinfection before returning to full susceptibility.

Passive screening is implemented as a constant stage-specific rate of case reporting and treatment. Active screening and treatment are implemented as an impulse control in the model, assumed to occur during the beginning of the year and all patients testing positive were treated. Active screening coverage was informed by the data. Initial conditions were taken as endemic equilibria in the absence of active screening or vector control as it was assumed that passive screening was the only intervention available before 2000.

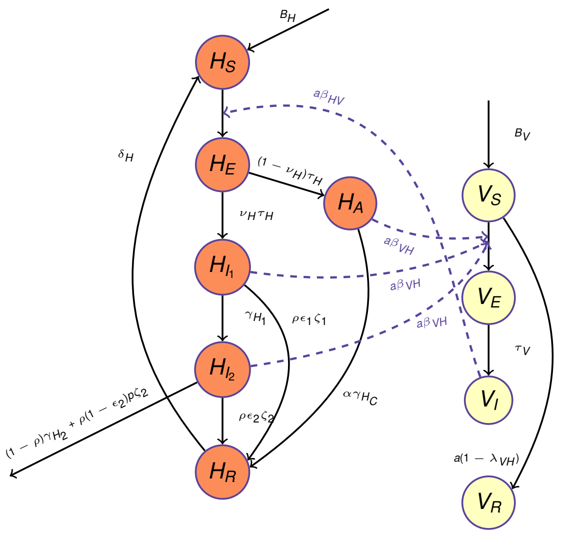

Figure 13: Schematic of Model Y. The figure is reproduced from [8], and shows disease transmission and progression for human and tsetse flies populations. Here the human population is divided into H_S, susceptible, H_E, exposed, H_A, asymptomatic carriers, H_{I1}, stage 1 infection, H_{I2}, stage 2 infection, and H_R, recovered. Tsetse population is divided into V_S, susceptible, V_E, exposed, V_I, infected, and V_R, recovered. Solid arrows represent the flow of individuals in and out of the different compartments, and the dashed lines represent the role of the different infected compartments on disease transmission.

Fitting procedure

In the previous study, our model was fitted using a Bayesian melding approach ([8]). Here, we fitted the model to passive and active data from the former Bandundu province of DRC using an approximate Bayesian computation Markov chain Monte Carlo (ABC-MCMC) with a Metropolis-Hastings sampling method. We assumed that only a proportion of inhabitants of Bandundu province are a risk of infection, and we estimated that proportion through model fitting. We also assumed that a proportion of individuals with stage 2 infection would not be identified/reported.

Eight parameters (Table 9) were fitted including the vector-to-human ratio (r_{VH}), rates of passive detection for stage 1 (ζ_1) and stage 2 (ζ_2) infections, the probability of developing symptomatic infection (ν_H), relative duration of asymptomatic infection compare to combined duration of stage 1 and 2 (σ_A), basic reproductive number (R_0), the proportion of the population at risk of infection (P_{risk}), and proportion of stage 2 cases under-reported (κ). The basic reproductive number was computed using the next generation matrix approach, and was used to compute the transmission probability from tsetse to humans.

We used the relative distance between data and model outcomes, Δ as a measure of error in the ABC-MCMC routine, with $\Delta = \sum_i ((D_i - M_i)/D_i)^2$ for reported cases from data D_i and reported cases from the model M_i in year i. We considered different tolerance values varying from 0.5 to 15, and identified the value 5 as the lowest value for which convergence was achieved after 500,000 MCMC steps with a burn-in of 50,000 steps. The Gelman-Rubin diagnostic was used to test for convergence of the MCMC chains [20]. Summary statistics and plots of the posterior distributions are given in Table 10 and Figures 14–16, respectively.

Notation	Description	Value	Reference
H	Total human population size	Varies	[5]
$1/\mu_H$	Life expectancy	59 years	[21]
B_H	Human population growth rate	calibrated to H	[21]
B_V	Tsetse constant birth rate	0.05/day	[22]
μ_V	Tsetse death rate	B_V	-
$1/\sigma_V$	Susceptibility period in tsetse	1 day	[22]
a	Tsetse biting rate	0.333/day	[22]
β_{HV}	Transmission probability from tsetse to humans	Derived from R_0	-
$1/\tau_V$	Incubation period in tsetse	25 days	[22]
$1/\tau_H$	Incubation period in humans	12 days	[22]
$1/\gamma_{H1}$	Stage 1 infectious period without treatment	526 days	[23]
$1/\gamma_{H2}$	Stage 2 infectious period without treatment	252 days	[13]
$1/\delta_H$	Immune period in humans after treatment	50 days	[22]
β_{VH}	Transmission probability from humans to tsetse	0.065	[22, 24]
ρ	Probability that a HAT patients gets a positive CATT and then a positive antibody/Trypanolysis test	0.87	[25]
ϵ_1	Efficacy of stage 1 treatment (pentamidine)	0.94	[26]
ϵ_2	Efficacy of stage 2 treatment (nifurtimox-eflornithine)	0.965	[27]
p	Probability of death due to stage 2 treatment failure (nifurtimox-eflornithine)	0.007	[27]

Table 8: Parameter description for Model Y.

Notation	Description	Unit	Prior
P_{risk}	Proportion of the human population at risk of infection	-	$X \sim$ Unif$[0.2, 1]$
R_0	Basic reproductive number	-	$X \sim$ Unif$[1, 3]$
ν_H	Probability of developing symptomatic infection	-	$X \sim$ Unif$[0.6, 1]$
r_{VH}	Tsetse-to-human ratio	-	$X \sim$ Unif$[1, 50]$
σ_A	Relative infectious period of asymptomatic carriers	-	$X \sim$ Unif$[1, 5]$
ζ_1	Treatment seeking rate of stage 1 patients	Year^{-1}	$X \sim$ Unif$[0, 1]$
ζ_2	Treatment seeking rate of stage 2 patients	Year^{-1}	$X \sim$ Unif$[0, 5]$
κ	Proportion of stage 2 infection undetected	-	$X \sim$ Unif$[0, 0.5]$

Table 9: Priors for fitted parameters for Model Y.

Parameter	Fit A		Fit B		Fit C	
	Median	95% CI	Median	95% CI	Median	95% CI
R_0	1.24	[1.11,1.51]	1.17	[1.09, 1.52]	1.23	[1.12, 1.52]
ν_H	0.73	[0.6, 0.9]	0.7	[0.6, 0.85]	0.71	[0.6, 0.84]
τ_1	0.18	[0.01, 0.4]	0.06	[0.03, 0.11]	0.045	[0.034, 0.059]
τ_2	0.22	[0.12, 0.45]	0.70	[0.24, 1.16]	0.46	[0.22, 0.78]
r_{VH}	25.6	[1.0, 49.2]	31.09	[30.7, 31.5]	31.6	[31.2, 31.8]
P_{risk}	041	[0.21, 0.95]	0.42	[0.23, 0.87]	0.63	[0.43, 0.82]
κ	0.15	[0.05, 0.49]	0.17	[0.01, 0.47]	0.23	[0.02, 0.49]
σ_A	8.7	[3.3, 14.4]	6.0	[3.2, 12.1]	7.8	[2.6, 14.9]

Table 10: Summary statistics of the posterior distributions for parameters for Model Y.

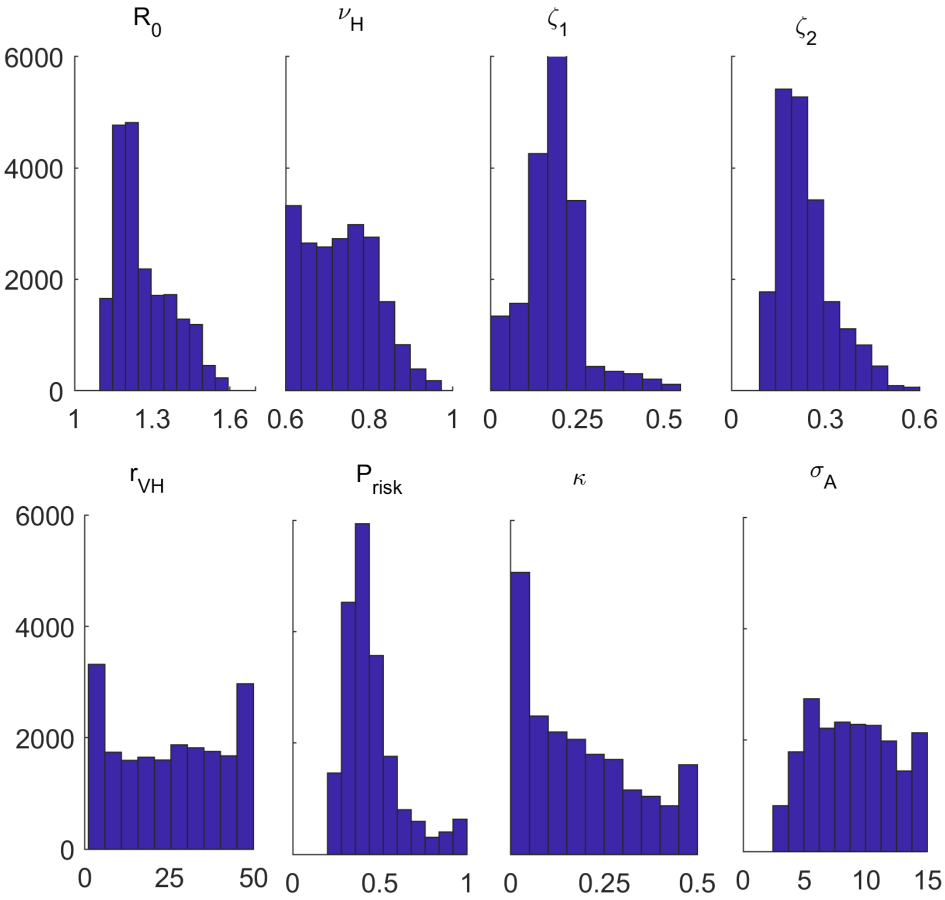

Figure 14: Posterior distributions of Fit A for Model Y.

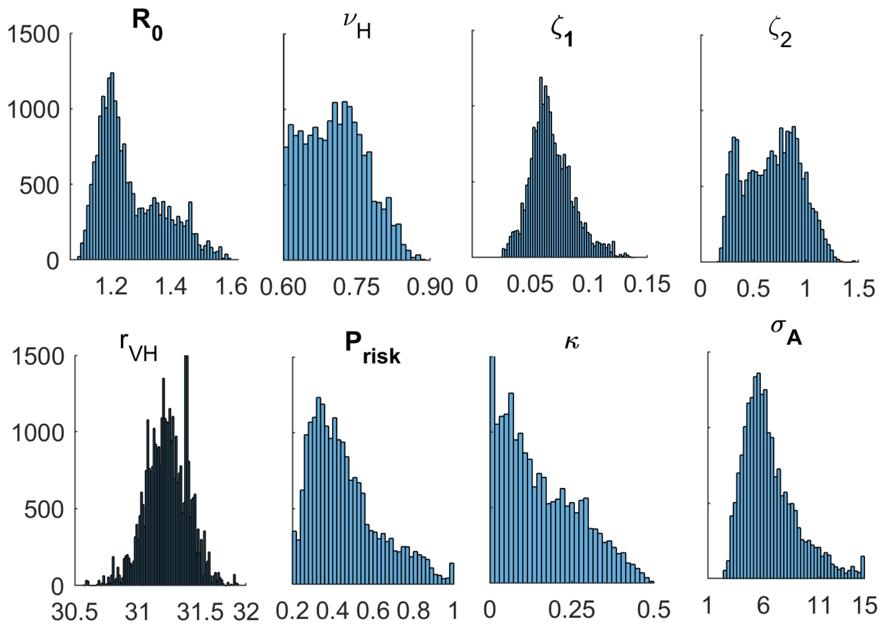

Figure 15: Posterior distributions of Fit B for Model Y. The same prior distributions as in Fit A was used.

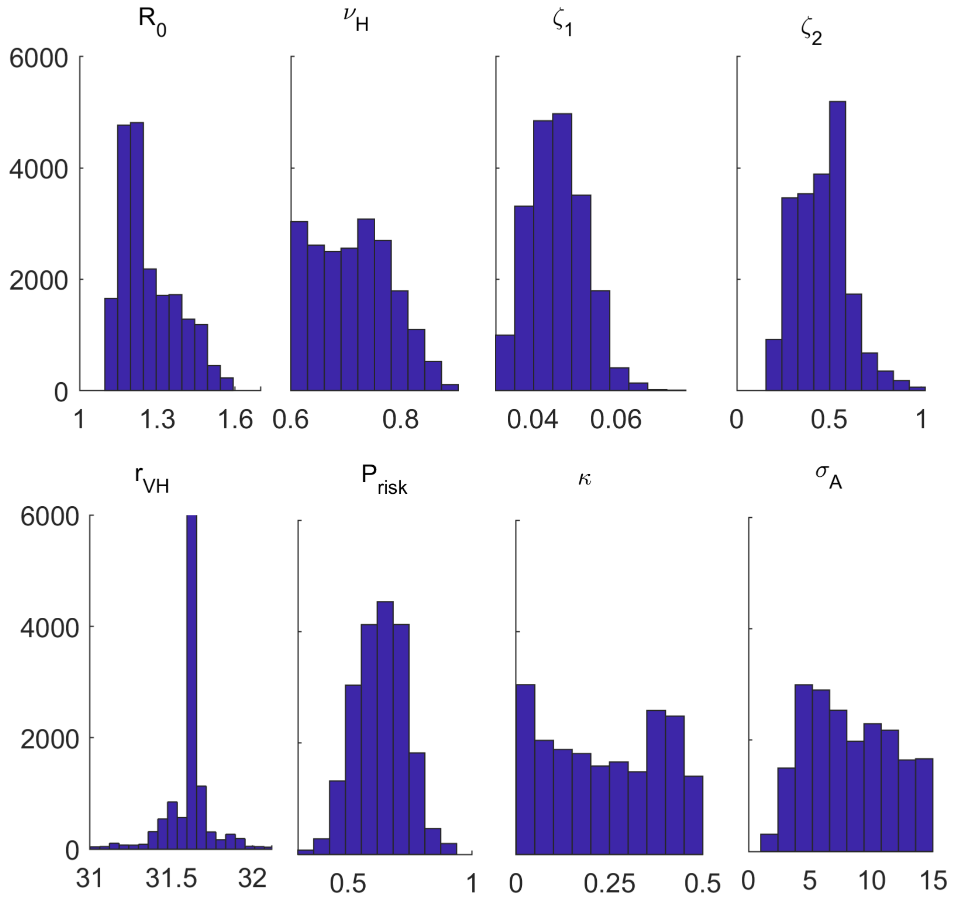

Figure 16: Posterior distributions of Fit C for Model Y. The same prior distributions as in Fit A was used.

Bibliography

[1] Rock KS, Ndeffo-Mbah ML, Castaño S, Palmer C, Pandey A, Atkins KE, et al. Assessing strategies against Gambiense sleeping sickness through mathematical modeling. Clinical infectious diseases. 2018;66(suppl_4):S286–S292.

[2] Dyer NA, Rose C, Ejeh NO, Acosta-Serrano A. Flying tryps: survival and maturation of trypanosomes in tsetse flies. Trends in parasitology. 2013;29(4):188–196.

[3] Stone CM, Chitnis N. Implications of heterogeneous biting exposure and animal hosts on Trypanosomiasis brucei gambiense transmission and control. PLoS computational biology. 2015;11(10):e1004514.

[4] Artzrouni M, Gouteux JP. A compartmental model of sleeping sickness in central Africa. Journal of Biological Systems. 1996;4(04):459–477.

[5] Institut National de la Statistique, Ministere du Plan et Revolution de la Modernite de la Republique Democratique du Congo. Annuaire statistique 2014; 2015. http://www.ins-rdc.org.

[6] Rock KS, Torr SJ, Lumbala C, Keeling MJ. Quantitative evaluation of the strategy to eliminate human African trypanosomiasis in the Democratic Republic of Congo. Parasites & vectors. 2015;8(1):532.

[7] Rock KS, Pandey A, Ndeffo-Mbah M, Atkins K, Lumbala C, Galvani A, et al. Data-driven models to predict the elimination of sleeping sickness in former Equateur province of DRC. Epidemics. 2017;18:101–112.

[8] Rock KS, Torr SJ, Lumbala C, Keeling MJ. Predicting the impact of intervention strategies for sleeping sickness in two high-endemicity health zones of the Democratic Republic of Congo. PLoS neglected tropical diseases. 2017;11(1):e0005162.

[9] Mahamat MH, Peka M, Rayaisse JB, Rock KS, Toko MA, Darnas J, et al. Adding tsetse control to medical activities contributes to decreasing transmission of sleeping sickness in the Mandoul focus (Chad). PLoS neglected tropical diseases. 2017;11(7):e0005792.

[10] The World Bank. Data:Democratic Republic of Congo; Accessed 2015. Available from: `http://data.worldbank.org/country/congo-democratic-republic`.

[11] Rogers DJ. A general model for the African trypanosomiases. Parasitology. 1988;97:193–212.

[12] Checchi F, Filipe JAN, Barrett MP, Chandramohan D. The natural progression of Gambiense sleeping sickness: What is the evidence? PLoS Neglected Tropical Diseases. 2008;2(12):e303.

[13] Checchi F, Funk S, Chandramohan D, Haydon DT, Chappuis F. Updated estimate of the duration of the meningo-encephalitic stage in gambiense human African trypanosomiasis. BMC Research Notes. 2015;8(1):292.

[14] Mpanya A, Hendrickx D, Vuna M, Kanyinda A, Lumbala C, Tshilombo V, et al. Should I Get Screened for Sleeping Sickness? A Qualitative Study in Kasai Province, Democratic Republic of Congo. PLoS Neglected Tropical Diseases. 2012;6(1):e1467.

[15] Checchi F, Chappuis F, Karunakara U, Priotto G, Chandramohan D. Accuracy of Five Algorithms to Diagnose Gambiense Human African Trypanosomiasis. PLoS Neglected Tropical Diseases. 2011;5(7):e1233–15.

[16] Davis S, Aksoy S, Galvani AP. A global sensitivity analysis for African sleeping sickness. Parasitology. 2010;138(04):516–526.

[17] Ravel S, Grebaut P, Cuisance D, Cuny G. Monitoring the developmental status of Trypanosoma brucei gambiense in the tsetse fly by means of PCR analysis of anal and saliva drops. Acta Tropica. 2003;88(2):161–165.

[18] WHO. Control and surveillance of human African trypanosomiasis; 2013. 984.

[19] Clausen PH, Adeyemi I, Bauer B, Breloeer M, Salchow F, Staak C. Host preferences of tsetse (Diptera: Glossinidae) based on bloodmeal identifications. Medical and Veterinary Entomology. 1998;12(2):169–180.

[20] Brooks SP, Gelman A. General methods for monitoring convergence of iterative simulations. Journal of computational and graphical statistics. 1998;7(4):434–455.

[21] The World Factbook. Country comparison: Life expectancy at birth; Accessed 12 August 2015. Available from: https://www.cia.gov/library/publications/the-world-factbook/rankorder/2102rank.html.

[22] Rogers D. A general model for the African trypanosomiases. Parasitology. 1988;97(01):193–212.

[23] Checchi F, Filipe JA, Haydon DT, Chandramohan D, Chappuis F. Estimates of the duration of the early and late stage of gambiense sleeping sickness. BMC infectious diseases. 2008;8(1):1.

[24] Davis S, Aksoy S, Galvani A. A global sensitivity analysis for African sleeping sickness. Parasitology. 2011;138(04):516–526.

[25] Brun R, Blum J, Chappuis F, Burri C. Human african trypanosomiasis. The Lancet. 2010;375(9709):148–159.

[26] Doua F, Miezan T, Sanon SJ, Boa YF, Baltz T. The efficacy of pentamidine in the treatment of early-late stage Trypanosoma brucei gambiense trypanosomiasis. The American journal of tropical medicine and hygiene. 1996;55(6):586–588.

[27] Priotto G, Kasparian S, Mutombo W, Ngouama D, Ghorashian S, Arnold U, et al. Nifurtimox-eflornithine combination therapy for second-stage African Trypanosoma brucei gambiense trypanosomiasis: a multicentre, randomised, phase III, non-inferiority trial. The Lancet. 2009;374(9683):56–64.
