# Supplementary material for: Assessing the impact of aggregating disease stage data in model predictions of human African trypanosomiasis transmission and control activities in Bandundu province (DRC)
Source: PLoS Negl Trop Dis. 2020 Jan 21;14(1):e0007976. doi: 10.1371/journal.pntd.0007976 (PMC6994134; doi:10.1371/journal.pntd.0007976)
Supplement: S3 Text — Projections on the annual incidence of new infections for all combinations of models and data sets. (PDF) [file pntd.0007976.s003.pdf]

S3 Text. Projections on new infections

Here we show the predicted underlying infection dynamics for each of the models under the three fits and for strategies including baseline medical intervention, vector control and enhanced passive detection. Fits for the subset staged data only used 2000-2006 data, so projections run from 2007 onwards, in contrast to the other projection which begin in 2013, after the data set ends. Predicted transmission is used to assess achievement of the 2030 elimination target.

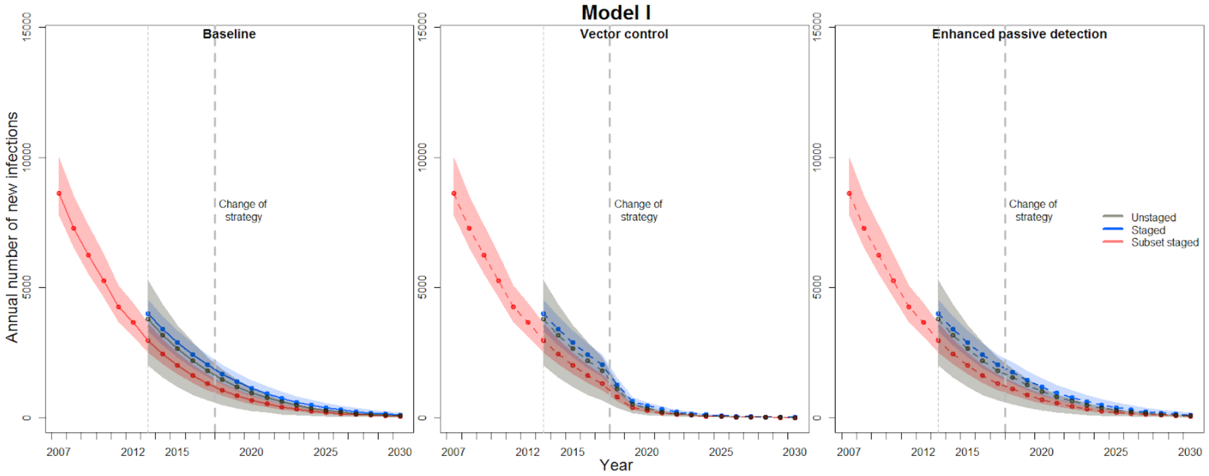

Figure A. Projections on the annual incidence of new infections under different fit for Model I. The points represent the median number of annual new infections with the associated shadow representing the 95% CI (credible interval). The vertical line in year 2013 indicates start of projections for fits to unstaged and staged datasets.

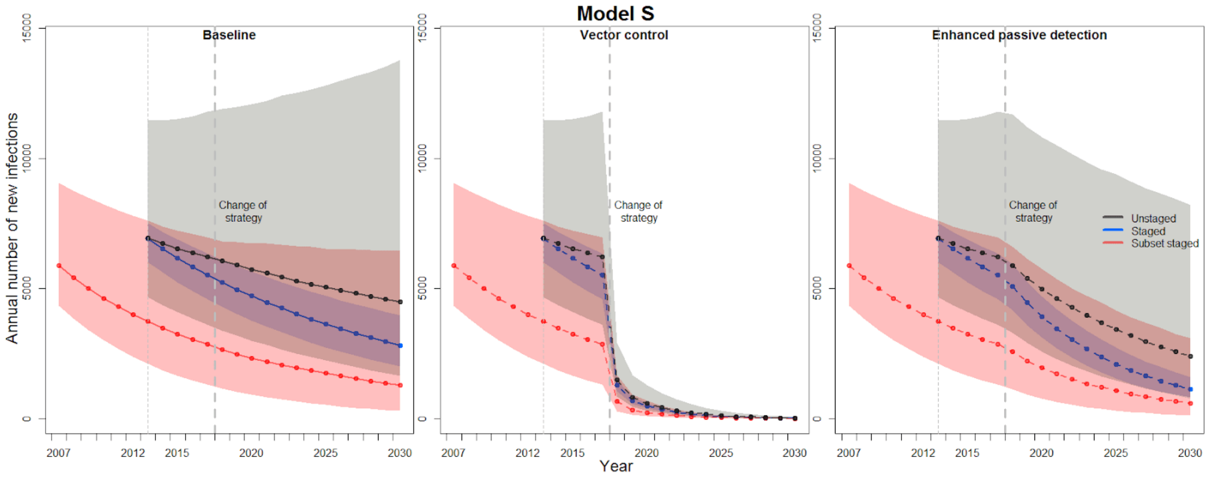

Figure B. Projections on the annual incidence of new infections under different fit for Model S. The points represent the median number of annual new infections with the associated shadow representing the 95% CI. The vertical line in year 2013 indicates start of projections for fits to unstaged and staged datasets.

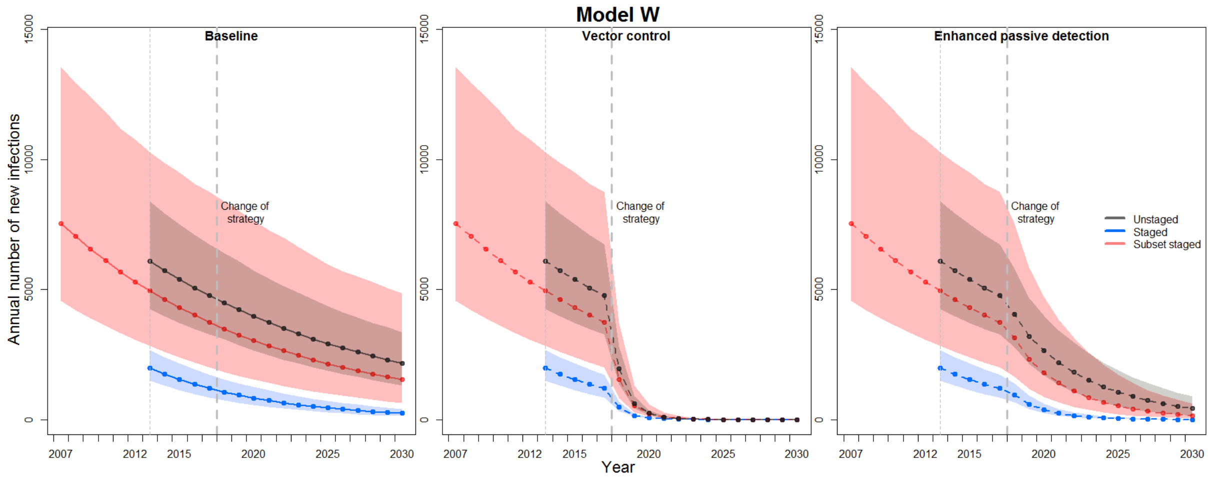

Figure C. Projections on the annual incidence of new infections under different fit for Model W. The points represent the median number of annual new infections with the associated shadow representing the 95% CI. The vertical line in year 2013 indicates start of projections for fits to unstaged and staged datasets.

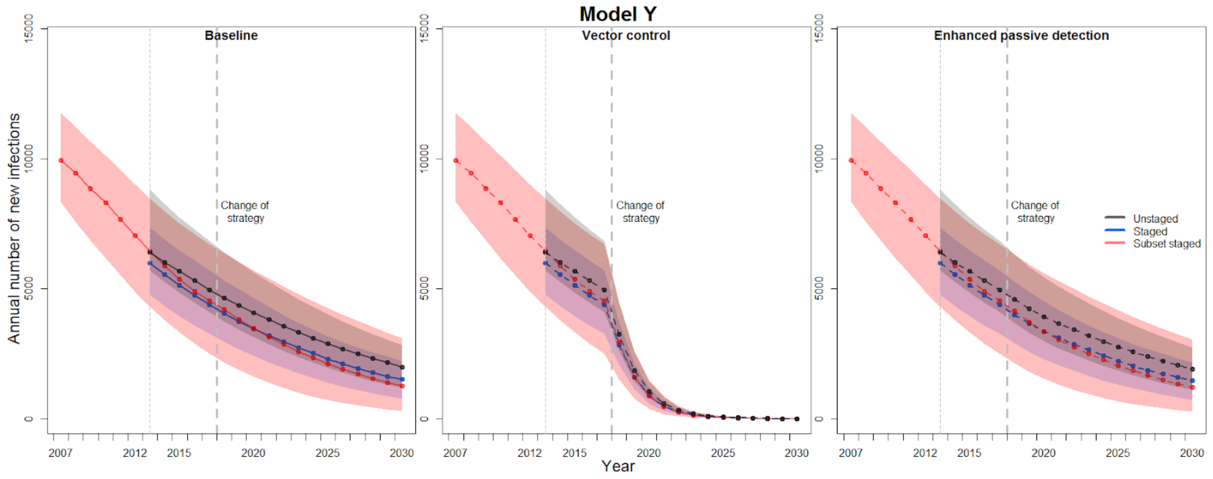

Figure D. Projections on the annual incidence of new infections under different fit for Model Y. The points represent the median number of annual new infections with the associated shadow representing the 95% CI. The vertical line in year 2013 indicates start of projections for fits to unstaged and staged datasets.

Averted new infections

Here we show the percentage of averted additional new cases under the two intensified interventions (e.g. vector control and enhanced passive detection) relative to the number of new infections predicted for the baseline, for the three different fits considered.

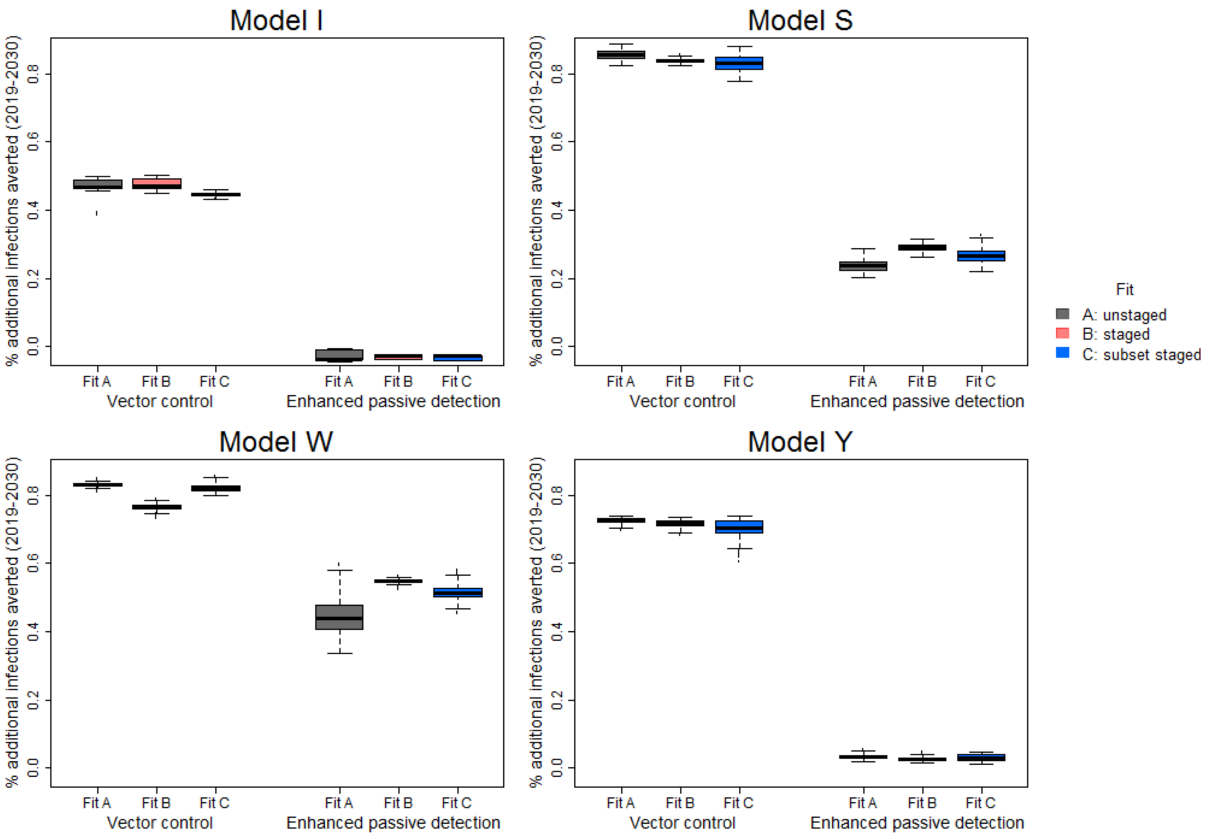

Figure E. Percentage of new infections averted between 2018 and 2030 for the intensified strategies explored here (vector control and enhanced passive surveillance) when compared to the baseline strategy under different fits explored. By showing proportions instead of absolute values, we indirectly include the variability among fits for a given model in the number of new infections expected. The negative cases averted in Model I are due to the fact that the enhanced passive screening increases the pool of susceptible individuals, which leads to a corresponding rise in the number of new cases.
