# Supplementary material for: Assessing the impact of aggregating disease stage data in model predictions of human African trypanosomiasis transmission and control activities in Bandundu province (DRC)
Source: PLoS Negl Trop Dis. 2020 Jan 21;14(1):e0007976. doi: 10.1371/journal.pntd.0007976 (PMC6994134; doi:10.1371/journal.pntd.0007976)
Supplement: S4 Text — Projections on the annual HAT cases for all combinations of models and data sets. (PDF) [file pntd.0007976.s004.pdf]

S4 Text. Projections on case reporting

In Figure 1 of the main text we show the estimation of reported cases compared to data used for fitting. In this section we display the corresponding expected reporting projections for different fits up to 2030. Note that the reported cases may be lower than the new infections due to underreporting, however improvements to passive detection increase reporting whilst decreasing transmission.

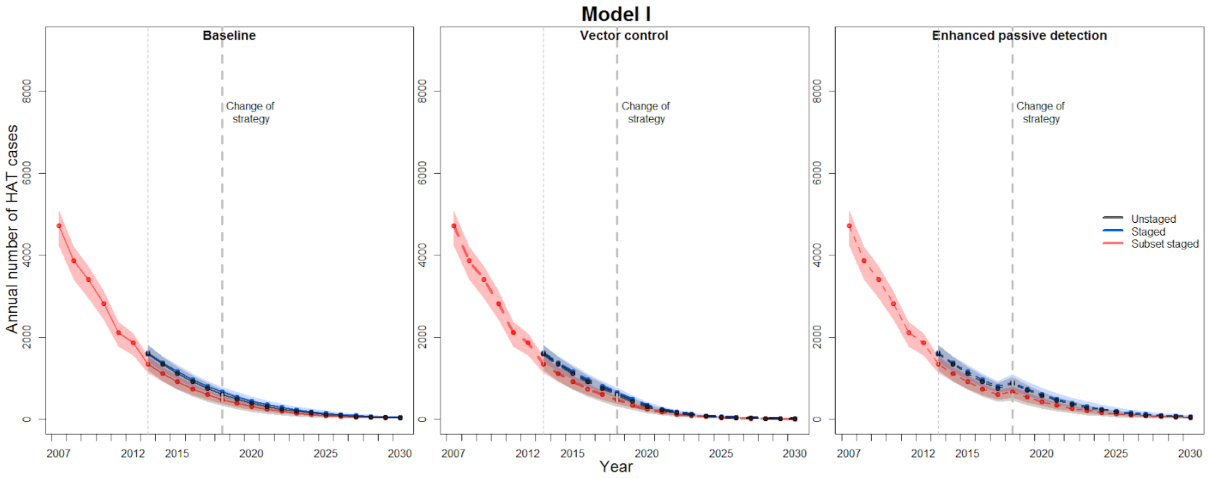

Figure A. Projections on the annual HAT cases under different fit for Model I. The points represent the median number of reported cases with the associated shadow representing the 95% CI (credible intervals). The vertical line in year 2013 indicates start of projections for fits to unstaged and staged datasets.

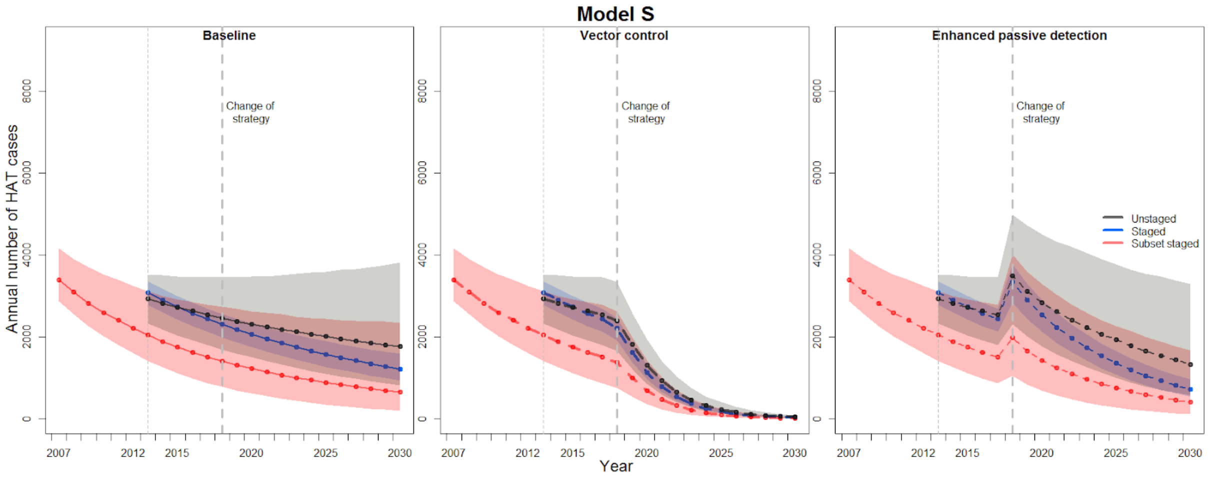

Figure B. Projections on the annual HAT cases under different fit for Model S. The points represent the median number of reported cases with the associated shadow representing the 95% CI. The vertical line in year 2013 indicates start of projections for fits to unstaged and staged datasets.

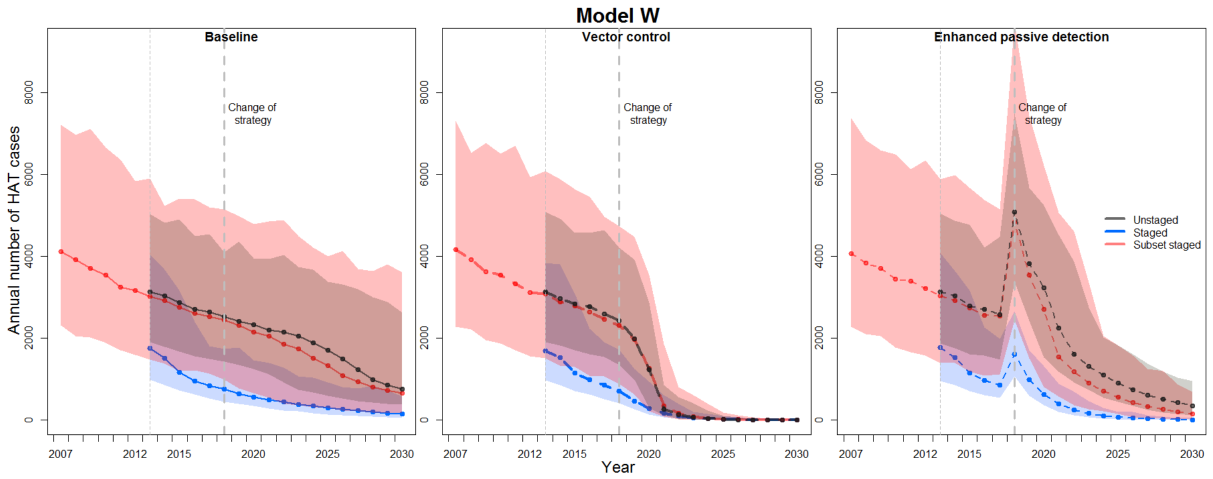

Figure C. Projections on the annual HAT cases under different fit for Model W. The points represent the median number of reported cases with the associated shadow representing the 95% CI. The vertical line in year 2013 indicates start of projections for fits to unstaged and staged datasets.

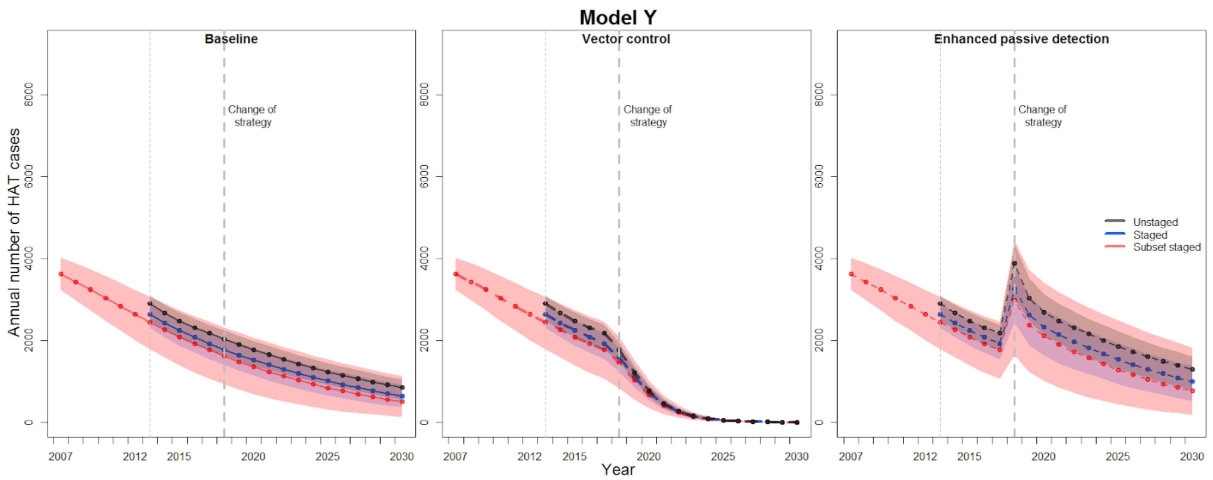

Figure D. Projections on the annual HAT cases under different fit for Model Y. The points represent the median number of reported cases with the associated shadow representing the 95% CI. The vertical line in year 2013 indicates start of projections for fits to unstaged and staged datasets.
