# Supplementary material for: Assessing the impact of aggregating disease stage data in model predictions of human African trypanosomiasis transmission and control activities in Bandundu province (DRC)
Source: PLoS Negl Trop Dis. 2020 Jan 21;14(1):e0007976. doi: 10.1371/journal.pntd.0007976 (PMC6994134; doi:10.1371/journal.pntd.0007976)
Supplement: S1 Table — (PDF) [file pntd.0007976.s005.pdf]

S1 Table. Probability of elimination (zero transmission) by 2030 with a weaker threshold

Table 3 in the main text gives the probability of elimination (zero transmission) by 2030 for the threshold of <1 new infection per 1,000,000. Due to the deterministic nature of all four models, a defined threshold is necessary since solutions approach zero but never reach zero. The table below shows the probability of achieving elimination using an alternative (weaker) threshold of <1 new transmission per 100,000. As expected for this threshold, there is a higher probability of the elimination target being met, although only Model I predicts elimination is likely with the baseline strategy. All models now predict elimination with vector control. Models S and Y predict that elimination is not feasible with enhanced passive detection (EPD), while Models I and W find elimination is possible in most of their fits.

	Strategy			Model
Fit	Baseline	Vector Control	EPD	
Unstaged	1	1	1	I
Staged	0.692	1	0.665	
Subset staged	1	1	1	
Unstaged	0	1	0	S
Staged	0	1	0	
Subset staged	0	1	0.05	
Unstaged	0	1	0.024	W
Staged	0.039	1	1	
Subset staged	0	1	0.474	
Unstaged	0	1	0	Y
Staged	0	1	0	
Subset staged	0.003	1	0.003	

Probability of different strategies achieving elimination by 2030 defined as <1 new transmission per 100,000 people. In all cases, 1000 simulations (i.e. different parameter sets) were used.
