# Supplementary figures and images for: Assessing the impact of aggregating disease stage data in model predictions of human African trypanosomiasis transmission and control activities in Bandundu province (DRC)
Source: PLoS Negl Trop Dis. 2020 Jan 21;14(1):e0007976. doi: 10.1371/journal.pntd.0007976 (PMC6994134; doi:10.1371/journal.pntd.0007976)

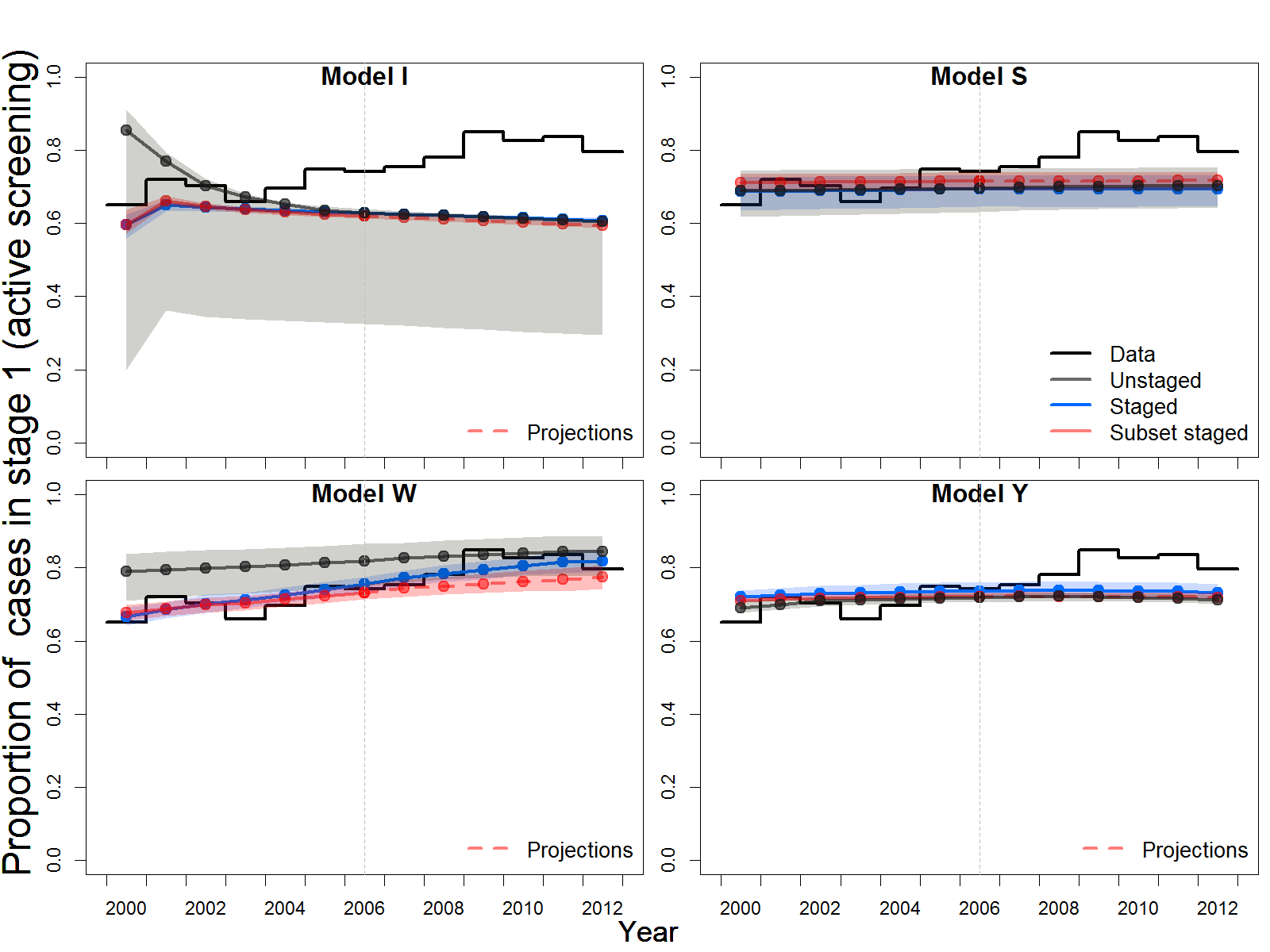

Supplement: S1 Fig — Proportion of stage 1 to total cases reported from active screening, and the corresponding estimation for a baseline strategy under different fitting. The posterior median is shown as a point. Dashed lines indicate projections based on fit to subset staged data. (PNG) [file pntd.0007976.s006.png]

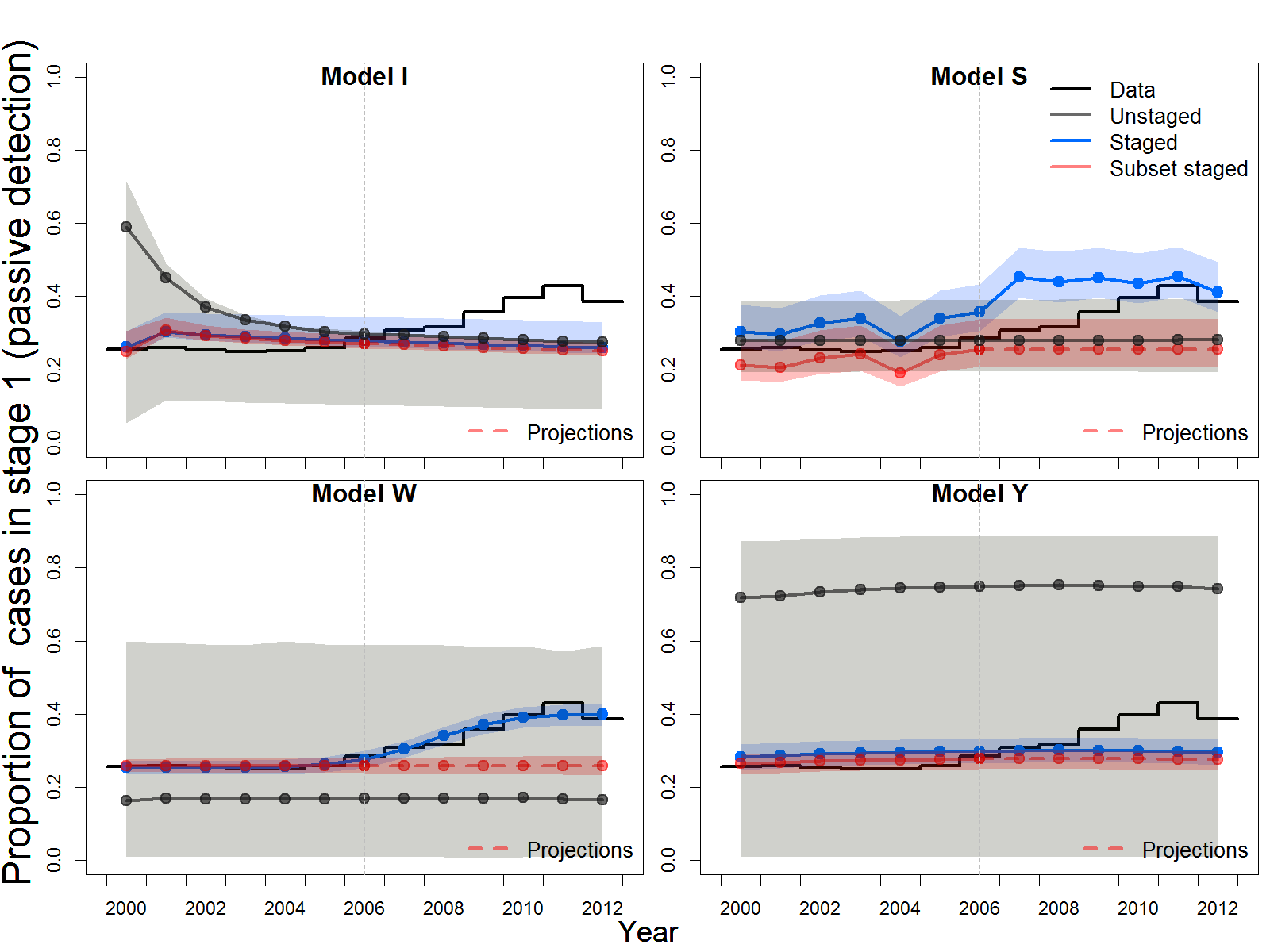

Supplement: S2 Fig — Proportion of stage 1 to total cases reported from passive detection, and the corresponding estimation for a baseline strategy under different fitting. The posterior median is shown as a point. Dashed lines indicate projections based on fit to subset staged data. (PNG) [file pntd.0007976.s007.png]
